# Lyotropic Liquid Crystal Mediated Assembly of Donor Polymers Enhances Efficiency and Stability of Blade‐Coated Organic Solar Cells

**DOI:** 10.1002/adma.202414632

**Published:** 2025-02-05

**Authors:** Azzaya Khasbaatar, Alec M. Damron, Pravini S. Fernando, Jasmine S. Williams, Chenhui Zhu, Eliot H. Gann, Jong‐Hoon Lee, Adrian Birge, Bora Kim, Sina Sabury, Minjoo L. Lee, John R. Reynolds, Ying Diao

**Affiliations:** ^1^ Department of Chemical and Biomolecular Engineering University of Illinois at Urbana‐Champaign 600 South Mathews Avenue Urbana IL 61801 USA; ^2^ Advanced Light Source Lawrence Berkeley National Laboratory Berkeley CA 94720 USA; ^3^ Materials Measurement Laboratory National Institute of Standards and Technology Gaithersburg MD 20899 USA; ^4^ Department of Advanced Materials Engineering Kyonggi University Suwon 16227 Republic of Korea; ^5^ Department of Materials Science and Engineering University of Illinois at Urbana‐Champaign 1304 W. Green St. Urbana IL 61801 USA; ^6^ Department of Electrical and Computer Engineering University of Illinois at Urbana‐Champaign 306 N. Wright St. Urbana IL 61801 USA; ^7^ School of Chemistry and Biochemistry Georgia Institute of Technology North Avenue Atlanta GA 30332 USA; ^8^ Beckman Institute University of Illinois at Urbana‐Champaign Urbana IL 61801 USA

**Keywords:** chiral symmetry breaking, liquid crystalline assembly, organic solar cells, polymer assembly, supramolecular chirality

## Abstract

Conjugated polymers can undergo complex, concentration‐dependent self‐assembly during solution processing, yet little is known about its impact on film morphology and device performance of organic solar cells. Herein, lyotropic liquid crystal (LLC) mediated assembly across multiple conjugated polymers is reported, which generally gives rise to improved device performance of blade‐coated non‐fullerene bulk heterojunction solar cells. Using D18 as a model system, the formation mechanism of LLC is unveiled employing solution X‐ray scattering and microscopic imaging tools: D18 first aggregates into semicrystalline nanofibers, then assemble into achiral nematic LLC which goes through symmetry breaking to yield a chiral twist‐bent LLC. The assembly pathway is driven by increasing solution concentration – a common driving force during evaporative assembly relevant to scalable manufacturing. This assembly pathway can be largely modulated by coating regimes to give 1) lyotropic liquid crystalline assembly in the evaporation regime and 2) random fiber aggregation pathway in the Landau–Levich regime. The chiral liquid crystalline assembly pathway resulted in films with crystallinity 2.63 times that of films from the random fiber aggregation pathway, significantly enhancing the T80 lifetime by 50‐fold. The generality of LLC‐mediated assembly and enhanced device performance is further validated using polythiophene and quinoxaline‐based donor polymers.

## Graphical abstract

This study reveals the concentration‐dependent self‐assembly of conjugated polymers, uncovering lyotropic liquid crystalline phases in several donor polymers. The extent of this self‐assembly process, determined by the solvent drying dynamics during blade coating, gives distinct film morphologies that significantly impact the device efficiency and stability, offering a framework for optimizing performance through precise control of coating conditions and polymer assembly.

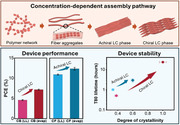

## 1 Introduction

Organic solar cells (OSCs) are a promising cost‐effective alternative to silicon solar cells owing to their ease of fabrication using solution‐processing techniques.^[^ ^1^, ^2^ ^]^ Their tunable molecular structures, inherent flexibility, and transparency also make them desirable for use in many emerging applications.^[^ ^3^, ^4^ ^]^ Over the past decade, design of novel photovoltaic materials have rapidly advanced OSCs to yield a commendable power conversion efficiency (PCE) exceeding 19%.^[^ ^5^, ^6^ ^]^ Nonetheless, one of the main bottlenecks remains the difficulty in controlling the bulk heterojunction (BHJ) morphology, particularly when using scalable coating techniques.^[^ ^7^, ^8^, ^9^ ^]^ BHJ‐based OSCs are comprised of an interpenetrating mixture of donor and acceptor materials, which form nanoscale phase separated morphology with crystalline donor and acceptor phases for efficient charge generation and transport.^[^ ^10^, ^11^, ^12^ ^]^ Other morphological parameters such as vertical phase separation, molecular orientation of the donor and acceptor materials can also influence the device performance.^[^ ^13^, ^14^, ^15^ ^]^ These morphological parameters depend strongly on the coating conditions, often resulting in time‐consuming trial‐and‐error‐based experiments to optimize the device performance of OSCs.^[^ ^16^, ^17^, ^18^ ^]^ Although high‐throughput experimentations coupled with machine learning techniques show promise in alleviating these efforts,^[^ ^19^, ^20^, ^21^ ^]^ a more general understanding of the structure‐property relationship remains to be elucidated.

Central to understanding the structure‐property relationship is the molecular assembly of conjugated systems, particularly conjugated polymers, from solution to solid‐state.^[^ ^22^ ^]^ During solution processing, conjugated polymers can undergo complex concentration‐dependent hierarchical self‐assembly that governs the film morphology and optoelectronic properties of organic electronic devices.^[^ ^22^, ^23^, ^24^ ^]^ Even at low concentrations, they can form various types of primary aggregates in their solution‐state depending on their molecular conformation, molecular weight, and solvent environment.^[^ ^18^, ^25^, ^26^, ^27^ ^]^ Besides forming primary aggregated structures, conjugated polymers may further assemble into secondary self‐assembled structures such as lyotropic liquid crystalline (LLC) mesophases and gel‐like networks.^[^ ^23^, ^28^, ^29^ ^]^ The LLC‐mediated assembly of conjugated polymers is of particular interest as numerous studies have shown that it can enhance the neat polymer film alignment, crystallinity, and molecular orientation, particularly at the air‐liquid interface.^[^ ^30^, ^31^ ^]^ Despite the significance of LLC‐mediated assembly on the neat film morphology, its impact on the blend or BHJ morphology of OSCs remains largely unexplored. So far, only a few studies have demonstrated the use of thermotropic liquid crystal (LC) molecules to improve the OSC performance, which was attributed to enhancing the film crystallinity.^[^ ^32^, ^33^, ^34^, ^35^ ^]^ Nevertheless, thermotropic LC phase transitions occur at temperatures far above the typical annealing temperature of OSCs, posing a challenge for their use in high‐performance systems. LLC‐mediated assembly of conjugated polymers, on the other hand, occurs upon simply increasing the solution concentration, which is inherent to evaporative solution processing. Therefore, elucidating the impact of LLC‐mediated assembly pathways on the blend film morphology and device properties of OSCs is essential for advancing the development of high‐performance solution‐processed OSCs.

Herein, we report the concentration‐dependent assembly pathway of a high performing conjugated donor polymer poly[(2,6‐(4,8‐bis(5‐(2‐ethylhexyl‐3‐fluoro)thiophen‐2‐yl)‐benzo[1,2‐b:4,5‐b′]dithiophene))‐alt‐5,5′‐(5,8‐bis(4‐(2‐butyloctyl)thiophen‐2‐yl)dithieno[3′,2′:3,4;2″,3″:5,6]benzo[1,2‐c][1,2,5]thiadiazole)] (D18), which undergoes formation of a LLC phase upon increasing the solution concentration. We show that D18 polymer forms semicrystalline fiber aggregates in the solution‐state that further assemble into achiral and chiral LLC phases sequentially as the solution concentration increases. It is further illustrated that the assembly pathway depends strongly on the coating conditions during meniscus‐guided coating, particularly the coating regime to give two distinct assembly pathways: 1) LLC mediated assembly in the evaporation regime and 2) random fiber aggregation in the Landau–Levich regime. The boiling point of the processing solvent also influences the extent of the LLC‐mediated assembly, leading to achiral and chiral LC‐mediated pathways, with the latter occurring when a high boiling point solvent is used. Upon fabrication of OSC devices, it is demonstrated that both achiral and chiral LC‐mediated assembly pathways enhance the PCE and stability of OSCs as compared to the random fiber aggregation pathway. In particular, achiral LC pathway improves the PCE by 20% and stability by three‐fold whereas the chiral LC pathway improves the PCE by 56% and T80 lifetime by 50‐fold compared to the random fiber aggregation pathway. We unveil that these improved OSC properties are attributable to the improved film crystallinity due to the formation of LLC phase in solution, which is achieved without compromising the domain sizes. Further, we show that the two assembly pathways defined here are general in two other polymer systems, poly[2,2⁗‐bis[[(2‐butyloctyl)oxy]carbonyl][2,2′:5′,2″:5″,2″‐quaterthiophene] ‐5,5‴‐diyl] (PDCBT) and poly [[6,7‐difluoro[(2‐hexyldecyl)oxy]‐5,8‐quinoxalinediyl]‐2,5‐thiophenediyl]] (PTQ‐10), and that the LLC pathway is overall conducive to improving performance in OPVs.

## 2 Results and Discussion

**Figure 1:**
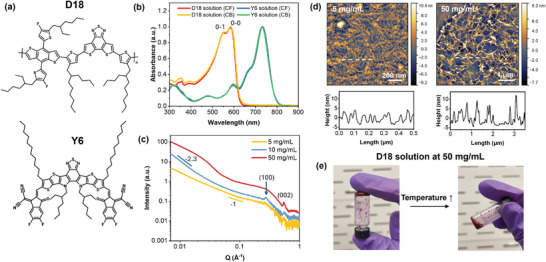
Solution state structure of D18. a) Molecular structures of the donor polymer D18 and small molecular acceptor Y6. b) UV–vis spectra of D18 and Y6 solutions at 5 and 8 mg mL^−1^ respectively prepared using chlorobenzene (CB) and chloroform (CF). c) Concentration‐dependent small angle X‐ray scattering (SAXS) profiles of D18 solution in CB. d) Freeze‐dried atomic force microscopy (AFM) height images of 5 and 50 mg mL^−1^ D18 solution in CB. e) Images of 50 mg mL^−1^ D18 solution in CB at room temperature before and after heating to 85 °C.

**Figure** **1**a shows the molecular structures of the conjugated donor polymer D18 and the small molecule acceptor 2,2′‐((2Z,2′Z)‐((12,13‐bis(2‐ethylhexyl)‐3,9‐diundecyl‐12,13‐dihydro‐[1,2,5]thiadiazolo[3,4‐e]thieno[2″,3″:4′,5′]thieno[2′,3′:4,5]pyrrolo[3,2‐g]thieno[2′,3′:4,5]thieno[3,2‐b]indole‐2,10‐diyl)bis(methanylylidene))bis(5,6‐difluoro‐3‐oxo‐2,3‐dihydro‐1H‐indene‐2,1‐diylidene))dimalononitrile (Y6) system used in this study. First reported by Liu et al., this system has been extensively investigated to obtain the record efficiency exceeding 19% in single junction solar cells.^[^ ^5^, ^36^ ^]^ However, it is important to note that such record‐breaking efficiencies are typically obtained from spin‐coated devices processed and tested entirely in controlled nitrogen environments, which are not representative of scalable or industrially relevant conditions. Notably, a recent study indicates that large‐area D18‐based solar cells processed using slot‐die coating achieve efficiencies of 12–13%, significantly lower than the 19.42% efficiency achieved by spin‐coated devices.^[^ ^37^ ^]^ Even devices fabricated using spin‐coating technique can vary significantly in different laboratories.^[^ ^38^ ^]^ These findings highlight the critical importance of understanding morphology control in OSCs, especially when transitioning to scalable coating methods. Our study, therefore, emphasizes the concentration‐dependent assembly pathway of D18 and its role in shaping the BHJ morphology and device properties of OSCs under scalable coating conditions, using the blade coating method.

We begin our investigation by first studying the solution‐state aggregation of D18. Figure 1b shows the UV–vis spectroscopic measurements of D18 and Y6 solutions in chlorobenzene (CB) and chloroform (CF), suggesting no observable difference in the spectroscopic characteristics of the solutions. In both solvents, D18 absorption spectra exhibit well‐defined vibronic features corresponding to 0–0 and 0–1 vibronic transitions, indicative of possible J‐aggregation with intrachain order in the solution‐state.^[^ ^39^ ^]^ Further complimentary absorption between D18 and Y6 is evident. To determine the structure of the polymer solution aggregates, small angle X‐ray scattering (SAXS) measurements were conducted on D18 solutions (CB) at 5, 10, and 50 mg mL^−1^ solution concentrations as shown in Figure 1c. The scattering profile of the 5 mg mL^−1^ solution is mostly featureless with a Porod decay of −1 in the Q range of Q < 0.1 Å^−1^, indicating rigid rod‐like polymer chains, consistent with its J‐aggregation behavior. At a Q ≈ 0.3 Å^−1^, a weak structure factor peak corresponding to correlations/associations between polymer chains is also observed. The existence of this structure factor peak indicates that polymers are aggregated, rather than existing as non‐interacting single polymer chains in the solution.^[^ ^40^ ^]^ Furthermore, atomic force microscopy (AFM) imaging of the freeze‐dried solution at 5 mg/mL concentration suggests polymer network‐like structures (Figure 1d) similar to our previous reports on other conjugated systems.^[^ ^18^, ^26^ ^]^ Since the network structure is not apparent from the solution scattering data, it is possible that the rod‐like polymer chains have not yet formed a network at 5 mg/mL and collapsed onto themselves during the freeze‐drying process. Further increasing the concentration to 10 mg/mL thus reveals a clear Porod decay in the low Q region (Q < 0.03 Å^−1^) with a slope of −2.3, denoting a branched fractal or network structure.^[^ ^41^ ^]^ The fact that the Porod slope is between −2 and −3 indicates mass fractal scattering rather than surface scattering (slope between −3 and −4).^[^ ^41^, ^42^ ^]^ This suggests that the polymer solution does not form large‐scale aggregates beyond the Q range, but rather become increasingly associated to form a fractal/network structures as the solution concentration increases.

Further increasing the solution concentration to 50 mg mL^−1^ leads to the formation of fibril aggregates as evidenced by the emergence of a Guinier knee in the low Q region (Q < 0.05 Å^−1^), corresponding to the cross‐section of the fibril aggregates (Figure 1c).^[^ ^18^ ^]^ Based on the double flexible cylinder model,^[^ ^40^ ^]^ the diameter of these fiber aggregates is determined to be 12.4 ± 0.6 nm (see Figure S4 and Table S1 (Supporting Information) for the deconvolution of the model fits and the resulting parameters), consistent with the AFM height profile of the freeze‐dried sample (Figure 1d). It is also observed that the solution becomes highly viscous and immobile at 50 mg/mL (Figure 1e) due to physical cross‐linking of the fiber aggregates in the solution‐state.^[^ ^28^ ^]^ Upon increasing the solution temperature, however, the solution becomes less viscous or more fluidic (Figure 1e) as the fiber aggregates start to dissolve at elevated solution temperatures evident from temperature‐variant SAXS (Figure S5, Supporting Information). We note that the (100) peak at 0.3 Å⁻¹ is absent in the 50 mg mL^−1^ solution. This is likely due to the experimental condition requiring heating of the gel‐like solution to enable its transfer to the measurement cell before cooling back to room temperature (see Experimental Methods for more information). As demonstrated in our previous study, this crystalline packing is highly sensitive to solution temperature and may require several hours to reform after disruption by thermal annealing.^[^ ^18^ ^]^

**Figure 2:**
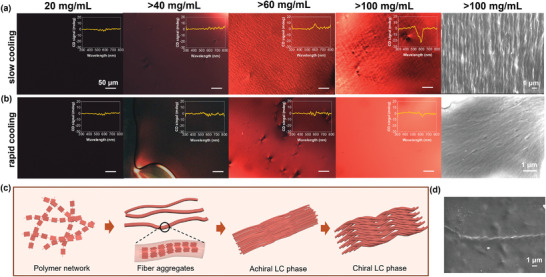
Concentration‐driven assembly pathways of D18. a) Cross‐polarized optical microscopy (CPOM) images of D18 solutions in chlorobenzene at various concentrations along with their corresponding CD spectra (images were taken at the edge of the solutions; thus, the actual concentrations are likely higher than the concentrations indicated) and the scanning electron microscopy (SEM) image of the freeze‐dried solution at >100 mg mL^−1^. CPOM images were taken by first heating the solution to 100 °C to reach the isotropic phase and then cooled down to room temperature at a rate of 10 °C min^−1^. b) CPOM images of the same D18 solutions prepared by rapidly quenching the solution to room temperature from the isotropic phase and the SEM image of the freeze‐dried solution at >100 mg mL^−1^. c) Schematic representations of concentration‐dependent assembly pathway of D18. d) SEM image of the large helical fiber observed in the bulk region of the >100 mg mL^−1^ freeze‐dried solution prepared by the slow cooling method described in part (a).

The concentration‐dependent assembly of D18 is further investigated using cross polarized optical microscopy (CPOM) and circular dichroism (CD) spectroscopy at solution concentrations up to ≈100 mg mL^−1^ using the drop‐and‐drying method^[^ ^23^ ^]^ (see details in Experimental Methods). Due to gelation/physical cross‐linking of D18 fibers at high concentrations (Figure 1e), the solutions were first heated up to 100 °C to reach the isotropic phase and then cooled down to room temperature at a cooling rate of 10 °C min^−1^ before taking CPOM and CD measurements. **Figure** **2**a shows the CPOM images of the D18 solutions (CB) at various concentrations along with their corresponding CD measurements (inset plots). CPOM and CD measurements of the D18 solutions in CF are provided in Figure S6 (Supporting Information), showing similar assembly behavior as CB solutions. Compared to the isotropic solution at 20 mg/mL, the solution at 40 mg mL^−1^ begins to display birefringence, implying polymer alignment attributed to the emergence of an LLC phase (Figure 2a). Upon increasing the solution concentration to 60 mg mL^−1^, striped periodic patterns emerge along with a weak but apparent CD signal, which further increases to give a dissymmetry factor (g‐factor) of 4 × 10^−4^ at 100 mg mL^−1^ solution concentration. This striped pattern resembles the twist‐bent chiral LC phase observed in other conjugated systems.^[^ ^23^, ^24^, ^43^, ^44^ ^]^ Consistently, scanning electron microscopy (SEM) imaging of the freeze‐dried solution at >100 mg mL^−1^ reveals that this chiral LC phase is comprised of micron‐scale helical fibers (Figure 2a). A clearer SEM image of a helical fiber taken in the bulk region of the sample shows a pitch length of 1.6 ± 0.2 µm (Figure 2d).

It is further demonstrated that this chiral LC phase succeeds the formation of an achiral LC phase. Video S1 (Supporting Information) illustrates the cooling process for an initial isotropic 60 mg mL^−1^ solution at high temperatures. During cooling, periodic striped patterns emerge from a featureless birefringent LC phase. By quenching the solution from the high‐temperature isotropic phase to room temperature, this featureless birefringent phase was preserved and confirmed to be achiral based on the CD measurement (Figure 2b). Freeze‐dried SEM imaging of this achiral LC phase at >100 mg mL^−1^ also reveals highly aligned nanoscale fibers (Figure 2c), distinct from the micron‐scale helical fibers formed in the chiral LC phase. This observation highlights that the chiral LC phase succeeds the formation of an achiral LC phase, as the nanofibers twist and bend to form the micron scale fibers at higher concentration or lower temperature. In fact, twist‐bent nematic LC phases are experimentally observed in achiral bent‐core molecules or bent mesogenic dimers.^[^ ^44^, ^45^ ^]^ Dozov predicted that negative bend elastic constant induces a local bend of the nematic director, stabilizing the twist‐bent chiral structures formed by these achiral bent‐shaped molecules.^[^ ^46^ ^]^ Although more commonly observed in bent‐core small molecules, chiral mesophases have also been observed in achiral conjugated polymer systems, such as PII‐2T and DPP‐based polymers, as reported in our previous works.^[^ ^23^, ^47^ ^]^ The emergence of chirality in these systems was attributed to their torsional polymer backbone conformation, leading to equal number of left‐ and right‐handed helices even at the molecular level, followed by asymmetric stacking/assembly of molecules which biases the population to one‐handedness at the macroscopic level.^[^ ^23^ ^]^ For D18, however, we observed that the supramolecular chiral assembly of D18 emerges from the helical assembly of achiral nanofibers in a very similar fashion that bent‐core shaped dimers form the twist‐bent mesophases. Therefore, we predict that the flexibility/shape of the nanofibers comprising the achiral LC phase of D18 lead to the emergence of the chiral LC formation upon increasing the solution concentration. We note that this is the first‐time chiral twist‐bend LC phase is observed in an organic solar cell system which has major implications on device properties shown below. In contrast to this multistep concentration‐dependent assembly pathway of D18, Y6 directly undergoes crystallization even at concentrations as low as ≈ 25 mg mL^−1^ (Figure S7, Supporting Information) upon reaching its solubility limit.

We next leveraged meniscus‐guided coating to control the assembly pathways of D18 to switch “on” or “off” the LC mesophase by tuning coating regimes. Recent works have shown that the coating regime during meniscus‐guided coating plays an important role on the assembly of conjugated polymers, and their resultant film morphology.^[^ ^47^, ^48^ ^]^ Depending on the coating speed and film thickness relationship, the coating regimes can be either in the evaporation or in the Landau–Levich (LL) regime.^[^ ^49^ ^]^ In the evaporation regime, the coating speed is slow enough that the film deposition is driven by the evaporation rate of the solvent. As a result, film thickness has an inverse relationship with the coating speed. In the LL regime, which occurs at high coating speeds, the film deposition is determined by the viscous shear force imparted by the blade. In between these two regimes occurs the transition regime, which results in the highest strain rate that can planarize the conjugated polymer backbone.^[^ ^47^ ^]^ However, coating in the transition regime often results in extremely thin films <50 nm, inappropriate for optimal performance of OSCs unless the solution concentration or the blade angle is dramatically increased. We thus limit our studies to comparing the evaporation and LL regimes using both CB and CF as the main solvents. Figure S8 (Supporting Information) shows an example of this film thickness versus coating speed relationship specifically for the D18:Y6 blend films processed from CF. The plot suggests that coating speeds below 0.7 mm s^−1^ fall within the evaporation regime, while coating speeds exceeding 15 mm s^−1^ correspond to the LL regime.

**Figure 3:**
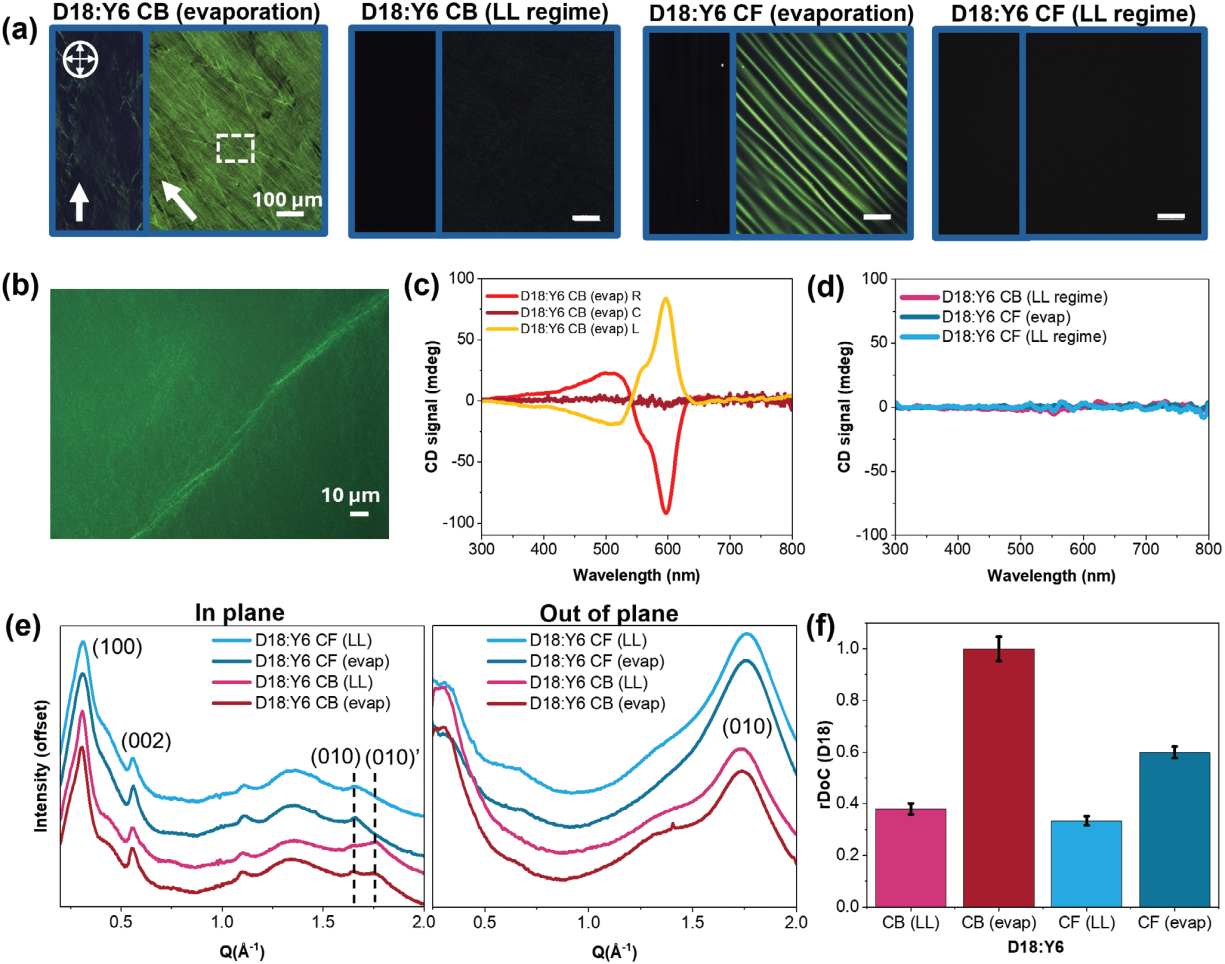
Assembly‐pathway‐dependent blend film morphology. a) CPOM images (10X magnification) of D18:Y6 (CB) and D18:Y6 (CF) blend films coated in the evaporation and Landau–Levich (LL) regimes. b) CPOM image (50X magnification) of the highlighted region with dashed lines in (a) showing formation of large helical fiber in D18:Y6 CB film coated in the evaporation regime. c) CD measurements of D18:Y6 CB coated in the evaporation regime taken at the right (R), center (C), and left (L) side of the film. d) CD measurements of D18:Y6 CB coated in the LL regime and D18:Y6 CF coated in both evaporation and LL regimes. e) In and out‐of‐plane sector profiles of the grazing incident wide‐angle X‐ray scattering (GIWAXS patterns of the blend films where (100) and (002) are the lamellar and backbone stacking peaks of the donor material and (010) and (010)’ peaks refer to the π–π stacking peaks of the donor and acceptor, respectively. f) relative degree of crystallinity (rDoC comparison of the blend films based on the (002) peak from D18. The error bars represent the combination of propagated error from curve fitting and film thickness variations.

Using CPOM imaging, we demonstrate that the blend films coated in the evaporation regime yield strong film alignment along the coating direction owing to the formation of LLC‐mediated assembly. **Figure** **3**a shows the CPOM images of the D18:Y6 blend films coated in two different regimes using CF and CB, respectively (the coating conditions are the same as the optimal device fabrication conditions). When coated in the evaporation regime at low coating speeds, D18:Y6 films yield highly birefringent films in both solvents, implying the formation of LC‐mediated pathways. However, when coated in the LL regime at high coating speeds, the blend films become isotropic without birefringence, indicating that the LC assembly is likely suppressed. Furthermore, the zoomed‐in CPOM image of the blend film deposited from CB solution in the evaporation regime reveals the existence of large helical fibers (Figure 3b), confirming the LC‐mediated assembly of D18. Interestingly, Figure 3c shows that the CD signal at the center of the film is weakly chiral whereas the left‐ and right‐hand side of the films exhibit g‐factors of 0.004 and −0.004, representing left and right‐handed helical fibers. This handedness inversion can be attributed to the flow direction during mesophase formation (see Figure S9, Supporting Information for the CPOM image of the films at the edge), which provides an asymmetric driving force for handedness selection.^[^ ^50^ ^]^ Without such asymmetric flow toward the edge of the film, it is expected that both helical senses appear with equal probabilities, thereby resulting in an overall weak CD signal at the center of the film.^[^ ^51^ ^]^ When coated in the LL regime or using CF as the main solvent, no CD signal was observed in any of these blend films (Figure 3d), although the film is birefringent when coated in the evaporation regime. This observation indicates that the assembly is kinetically suppressed due to the rapid evaporation of CF, providing insufficient time for achiral‐to‐chiral LC transition.

Grazing incident wide angle X‐ray scattering (GIWAXS) measurements further illustrate that coating regime (or assembly pathway) also influences the degree of crystallinity of the blend films. Shown in Figure 3e is the in‐ and out‐of‐plane sector profiles of the 2D GIWAXS patterns obtained for the blend films (Figure S10a, Supporting Information). Based on these plots, it is apparent that the blend films exhibit strong lamellar (100) and π–π stacking (010) peaks in the in‐ and out‐of‐plane directions, respectively. In the in‐plane direction, there are distinct (010) and (010)’ peaks from D18 and Y6, respectively in the films cast from CB, corresponding to π–π stacking distances of 3.81 and 3.56 Å. In blend films coated from CF, no in‐plane (010)’ peak from Y6 was observed, indicating the lack of edge‐on orientation of the acceptor material. Previous studies have already shown that the orientation of Y6 is largely dependent on the processing solvent.^[^ ^52^, ^53^ ^]^ Our neat film GIWAXS results are also consistent with these reports, suggesting that Y6 adopts more face‐on orientation in CF as compared to CB (Figure S10b, Supporting Information). In the out‐of‐plane direction, on the other hand, only one (010) peak was observed in both CB and CF blend films due to peak broadening. This (010) peak corresponds to π‐π stacking distances of 3.55 and 3.61 Å for CF and CB films, respectively. The π–π stacking distance of the CF blend film is closer to the neat acceptor π–π stacking distance at 3.53 Å, whereas that of the CB film is closer to the neat donor π–π stacking distance at 3.67 Å (Table S2, Supporting Information). The fact that CF blend film has a shorter out‐of‐plane π–π stacking distance also confirms that face‐on population of Y6 dominates the blend film coated from CF as compared to CB.

Besides the lamellar and π–π stacking peaks is a (002) peak related to the backbone ordering of D18 in the in‐plane direction, which enabled us to quantify the relative degree of crystallinity (rDoC) of D18 in the blend films.^[^ ^38^ ^]^ This peak also appears both in the neat D18 films (Figure S10b, Supporting Information) and in the 50 mg mL^−1^ solution based on our solution SAXS measurements (Figure 1c). The backbone order peak was thus used to determine the degree of crystallinity of the blend films since the π‐π stacking peaks are too convoluted to uniquely separate D18 and Y6 contributions, particularly in the out‐of‐plane direction. The rDoC values of the films were obtained by integrating the geometrically corrected intensities of the (002) peak and normalizing with the total illuminated area.^[^ ^54^ ^]^ The comparison of rDoC values (Figure 3f) suggest that the films coated in the evaporation regime exhibit higher crystallinity than those coated in the LL regime. In particular, the achiral and chiral LC‐mediated pathways produced films that are 1.82 and 2.63 times more crystalline than those from the random aggregation pathways. Interestingly, CB films coated in the evaporation regime exhibit the highest degree of crystallinity, suggesting a link between chiral helical assemblies and high crystallinity. Indeed, past works have shown that helical assemblies are thermodynamically driven to have the densest packing.^[^ ^55^, ^56^, ^57^ ^]^ Besides the coating regime, we also note that solvent has a drastic impact on the full width half maximum (FWHM) of the (010) and (100) peaks (Figure S12 and Table S2, Supporting Information) and the molecular orientation as previously discussed, which makes the D18:Y6 based OSCs highly sensitive to the processing solvent as previously reported in other works.^[^ ^52^, ^58^ ^]^

We attribute the enhanced film crystallinity and alignment of the films coated in the evaporation regime to the LLC mediated assembly of D18 during coating. Compared to the LL regime, where solvent drying occurs rapidly after a liquid layer is formed, coating in the evaporation regime forms a triple‐phase contact line which induces strong capillary flow toward the drying front.^[^ ^59^ ^]^ This process is known as the coffee ring effect, in which a spilled drop of coffee leaves dense, ring‐like deposit along the perimeter.^[^ ^60^ ^]^ Similarly, the capillary flow during coating in the evaporation regime results in concentration increase close to the contact line, which can induce the LC‐mediated assembly as shown in Figure 2.^[^ ^61^ ^]^ Additionally, the neat film GIWAXS measurements (Figures S11 and S12, Supporting Information) suggest that coating regime has a minimal influence on the crystal size of the neat films since the full‐width half maximum of the (010) and (100) peaks remain unchanged regardless of the coating regime. This suggests that the improved degree of crystallinity in blend films formed via the LLC‐mediated pathway is due to the formation of the increasing number of, rather than larger crystalline domains.

Based on the GIWAXS patterns shown in Figure S10 (Supporting Information), we also note that the blend films coated from CF in the evaporation regime show highly ordered out‐of‐plane peaks with at least four diffraction orders (excluding double diffractions) visible at 0.44, 0.55, 0.66, 0.77 Å^−1^, likely corresponding to fourth, fifth, sixth, and seventh order diffraction peaks with a first‐order peak at 0.11 Å^−1^. These peaks are also visible in D18 neat films cast from CF in the evaporation regime but not observed in neat films of Y6 (Figure S11, Supporting Information), suggesting that they originate from the donor rather than the acceptor material. The real space distance corresponding to this first‐order peak is 5.7 nm. These peaks are somewhat visible in the blend film‐coated from CB in the LL regime but absent in both neat and blend films coated from CB in the evaporation regime. These high‐order out‐of‐plane peaks have previously been attributed to alkyl sidechain phase forming vertically multilayered nanostructures.^[^ ^62^ ^]^ The fact that these peaks coexist with the alkyl sidechain stacking peak at 1.46 Å^−1^ confirms that they are related to the alkyl sidechain phase. We believe that such long‐range order alkyl sidechain phase may be suggestive of a smectic achiral LC phase kinetically quenched under the rapid solvent drying conditions since the films coated from CB under the evaporation regime (more thermodynamic deposition condition) is absent from this phase.

**Figure 4:**
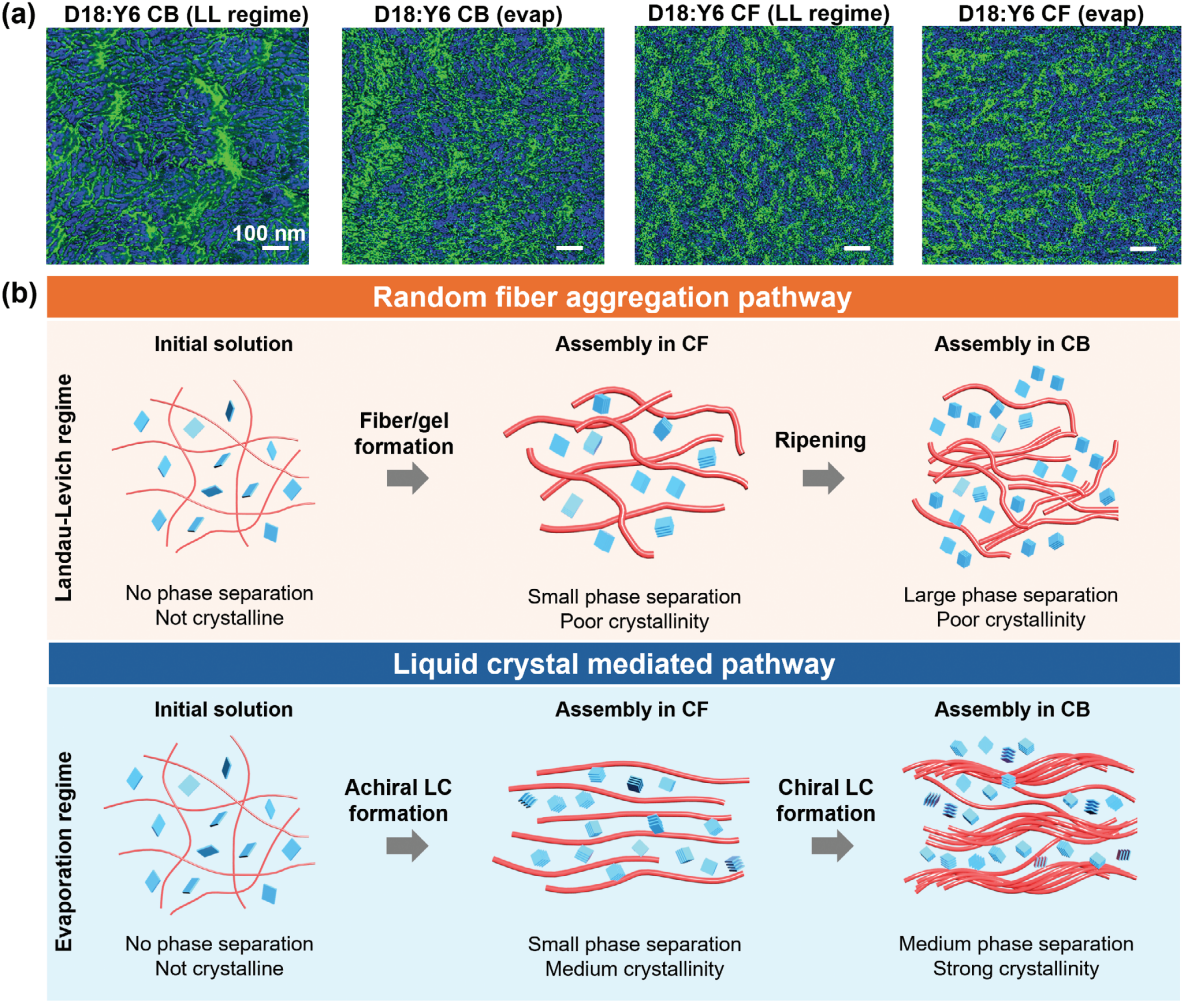
a) Photo‐induced Force Microscopy (PiFM) images of the blend films where the green and blue region represents the donor and acceptor domains, respectively. b) Proposed assembly pathways of D18 in the blend solution depending on the printing regimes. In the LL regime, D18 undergoes random fiber aggregation pathway whereas in the evaporation regime, it undergoes LC‐mediated assembly. Solvent determines the extent of assembly pathways in both cases, in which using CF results in kinetically quenched assembly with small phase separation and poor to medium crystallinity depending on the coating regimes. Using CB extends the film deposition time, leading to large‐scale phase separation.

To further investigate the assembly process in the blend, photo‐induced force microscopy (PiFM) was utilized to determine the phase‐separated structures of the blend films depending on the coating regimes. PiFM is a relatively novel scanning probe technique, which combines AFM with a tunable infrared (IR) laser for imaging the blend films of OSCs with chemical specificity.^[^ ^63^, ^64^ ^]^ Unlike IR‐based AFM, PiFM is conducted in noncontact mode, producing high‐resolution measurements in the sub‐10 nm range under ambient conditions.^[^ ^63^ ^]^ In recent years, PiFM has been successfully used in imaging the nanoscale phase‐separated blend film morphology of OSCs, enabling direct observation of the complex phase‐segregated BHJ morphology.^[^ ^65^, ^66^, ^67^ ^]^ **Figure** **4**a shows the false color overlayed PiFM images of the blend films (individual PiFM images representing only donor and acceptor domains before overlaying are provided in Figure S13, Supporting Information). The green and blue regions represent the donor and acceptor materials, respectively, each probed at wavelengths of 823 and 1437 cm^−1^ based on the IR spectra on the neat films (Figure S13a, Supporting Information). We note that the PiFM spectra of the neat films shown in Figure S13a (Supporting Information) resembles the bulk material spectra obtained from the fourier transform infrared (FTIR) spectroscopy (Figure S13b, Supporting Information).

The PiFM measurements (Figure 4a) suggest that the phase‐separated morphology depends intimately on the assembly pathways tuned by solvent and coating regimes. When coated in the LL regime from CB, D18:Y6 forms a blend morphology mainly dominated by the large acceptor domains that seem to have impeded percolation of donor domains. In contrast, coating in the evaporation regime reduces the sizes of acceptor domains due to the strong aggregation of D18 facilitated by the LLC mesophase formation. As outlined in our review paper,^[^ ^22^ ^]^ the domain size in miscible or partially miscible systems is determined by the competition between donor and acceptor crystallization. In this case, we propose that as the donor material undergoes enhanced aggregation and crystallization, the resulting increase in donor crystallinity limits the space available for the acceptor material to form its own crystalline domains. Simply put, as the donor material forms more crystalline domains, the acceptor material is constrained, leading to smaller, separate crystalline domains. The most drastic change occurs when changing the solvent from CB to CF, which results in significantly reduced donor and acceptor phase‐separated domain sizes regardless of the coating regimes. This substantial change in the phase‐separated structures upon changing the coating solvent is attributed in part to the kinetically quenched morphology when processing from CF. Interestingly, the coating regime has a marginal effect on the blend film morphology when a more volatile solvent CF is used, which was also confirmed using resonant soft X‐ray scattering (RSoXS) measurements (Figure S14, Supporting Information).

The film morphology characterization results allow us to propose the assembly pathways of D18 in the blend under different coating regimes and processing solvents as depicted in Figure 4b. In the LL regime, D18 undergoes random fiber aggregation as evidenced by the poor crystallinity and alignment of the blend films. If the assembly of D18 is allowed to proceed longer by using CB, the polymer fibers ripen further to give large D18 and Y6 domains. In contrast, when coated in the evaporation regime, D18 undergoes a LLC mediated pathway, forming either chiral or achiral LLC phases depending on the extent of assembly determined by the solvent drying time. When assembly is allowed to proceed further toward equilibrium with longer solvent drying time in CB, chiral LC forms by transitioning from the achiral LC which drives further aggregation of D18 to result in larger and more crystalline phase‐separated domains. Compared to the random aggregation, LLC mediated pathway, particularly chiral LC assembly, increases the crystallinity of D18 in the blend films. As a result, formation of chiral LC phase, compared to random aggregation of D18, reduces the large Y6 domains formed in the blend film. On the other hand, achiral LC assembly where the aggregation of D18 is kinetically suppressed by CF results in smaller‐scale phase separation that remains less sensitive to the coating regime.

**Figure 5:**
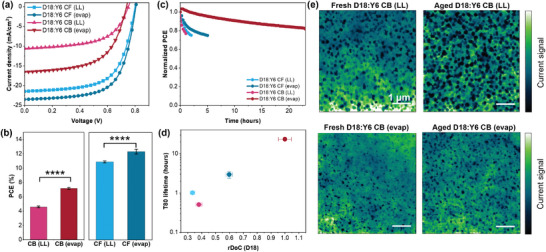
Assembly pathway‐dependent OSC device performance. a) Current density–voltage (*I–V*) characteristic curves and b) device performance comparison of D18:Y6 system fabricated in the two regimes from two different solvents. All statistical data are presented as mean ± standard deviation based on 8–11 devices (****p < 0.0001 indicate statistically significant differences between the samples). c) Device stability trend depending on the coating regime and solvent. d) T80 lifetime as a function of rDoC of D18 in the blend film. e) c‐AFM images of D18:Y6 blend films cast from CB in the evaporation and the LL regimes before and after light aging (1‐sun) for 24 h under N_2_ atmosphere.

Finally, OSC devices were fabricated using the conventional architecture (indium tin oxide (ITO)/poly(3,4‐ethylenedioxythiophene): poly(styrene‐sulfonate) (PEDOT:PSS)/D18:Y6/ N,N′‐Bis{3‐[3‐(Dimethylamino)propylamino]propyl}perylene‐3,4,9,10‐tetracarboxylic diimide (PDINN)/Ag) to determine how different assembly pathways and their resultant film morphology impact the device performance and stability. **Figure** **5**a,b shows the current density versus voltage relationship as well as the PCE comparison of devices fabricated from different solvents and coating regimes. The best PCE is obtained from CF in the evaporation regime reaching 12.3%, which is respectful given that the device is fully fabricated and tested in ambient conditions in glovebox free environment without any additive or post‐treatment. The device performance results (performance metrics are shown in **Table** **1**) suggest that the evaporation regime leads to OSCs with higher PCEs than those coated in the LL regime regardless of the solvent, which correlates well with the improved film crystallinity owing to the LC mediated pathways. Figure S15 (Supporting Information) shows that this trend holds true for a wide range of film thicknesses. Although devices coated in the evaporation regime exhibit higher PCEs in both solvents, we note that the difference is more pronounced in CB (Figure 5a,b). This observation can be attributed to the larger extent of domain size reduction and crystallinity enhancement of D18 in CB films coated in the evaporation regime as compared to the LL regime. As previously discussed, coating the film in the evaporation regime reduces the strong aggregation of Y6 in CB as D18 undergoes chiral LC‐mediated assembly which enhances the aggregation of the donor polymer (Figure 4a). This result thus suggests that chiral assembly can drastically improve the device performance by yielding denser packing and stronger crystallinity of the donor polymer, which reduces unfavorable aggregation of the acceptor.

**Table 1:** Photovoltaic parameters of D18:Y6, PDCBT:ITIC, and PTQ‐10:Y6 devices‐based OSCs under illumination of AM1.5G (100 mA cm^−2^) tested under ambient conditions in the air.

Sample	FF [%]	J_sc_ [mA cm^−2^]	V_oc_ [V]	PCE %
D18:Y6 CF (evap)	65.2 ± 0.9	23.5 ± 0.3	0.798 ± 0.002	12.3 ± 0.3
D18:Y6 CF (LL)	58.9 ± 2.2	21.7 ± 0.3	0.804 ± 0.003	10.2 ± 0.4
D18:Y6 CB (evap)	58.7 ± 1.6	16.6 ± 0.2	0.738 ± 0.005	7.2 ± 0.2
D18:Y6 CB (LL)	57.6 ± 0.4	10.6 ± 0.3	0.751 ± 0.004	4.6 ± 0.1
PDCBT:ITIC (Evap)	46.9 ± 0.9	19.4 ± 0.2	0.734 ± 0.001	6.7 ± 0.2
PDCBT:ITIC (LL)	52.3 ± 2.4	10.3 ± 0.1	0.860 ± 0.001	4.6 ± 0.2
PTQ‐10:Y6 (Evap)	73.1 ± 0.3	20.3 ± 0.2	0.814 ± 0.003	12.1 ± 0.1
PTQ‐10:Y6 (LL)	68.5 ± 0.4	20.1 ± 0.3	0.813 ± 0.005	11.2 ± 0.2

Furthermore, we quantified the device stability by T80 lifetime, which is the time it takes the devices to reduce to 80% of their initial performance under solar irradiation. We found that devices fabricated in the evaporation regime show significantly enhanced device stability (under nitrogen atmosphere) compared to those fabricated in the LL regime (Figure 5c). Specifically, chiral LC‐mediated assembly pathway (CB, evaporation regime) and achiral LC‐mediated pathway (CF, evaporation regime) improved the T80 lifetime by 50‐fold and 3‐fold, respectively, compared to the random fiber aggregation pathway (CB or CF, LL regime). We hypothesize that the assembly pathways dictate T80 by influencing the film crystallinity. As reported by Durrant et al., the crystallinity of the donor polymers impacts the triplet lifetime and the oxygen quenching yield, which determine the photochemical stability of OSCs.^[^ ^68^ ^]^ Indeed, we discover a strong, quantitative correlation between T80 and the relative degree of crystallinity of D18 in the blend film, with the chiral LC assembly pathway giving rise to the highest crystallinity and thus the highest T80 (Figure 5d). The enhanced T80 may come from either reduced photodegradation or more stable film morphology. Regarding photodegradation, we did not observe any changes in the UV‐Vis spectroscopy when comparing fresh and aged blend films under inert conditions (Figure S16, Supporting Information), suggesting that chemical degradation within the active layer is not the cause for device degradation in this case. To access morphological stability, we next compared the fresh and aged blend films using conductive atomic force microscope (c‐AFM) current mapping to determine how the blend film morphology changes upon solar irradiation. Figure 5e shows the c‐AFM hole current maps of the fresh and aged blend films cast from CB comparing the two coating regimes. Bright or higher hole current regions correspond to D18 domains, and darker or low hole current regions corresponds to Y6 domains.^[^ ^69^ ^]^ Based on this result, it is apparent that the blend film coated in the LL regime forms progressively larger Y6 domains with time, whereas the domain size change in film coated in the evaporation remains changes to a much less extent. We thus believe that chiral LC assembly pathway leads to enhanced crystallinity of D18 and improves the morphological stability of the OSCs by hindering the diffusion of Y6 material in the blend.

**Figure 6:**
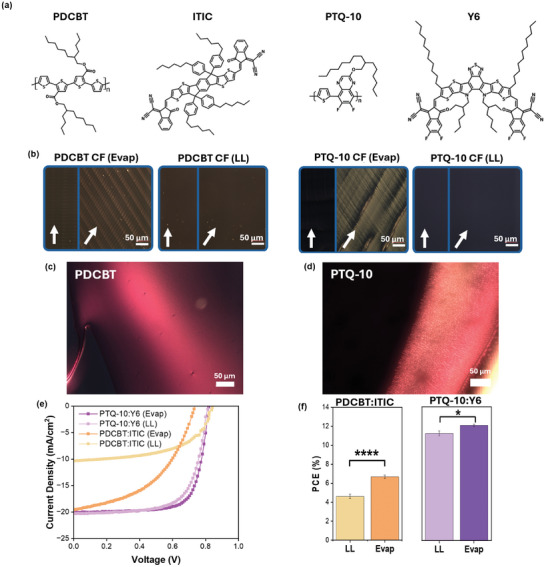
Generality of lyotropic liquid crystal (LLC) assembly pathway on performance of OPV conjugated polymers. a) Structures of polymer donors PDCBT and PTQ‐10, and nonfullerene acceptors ITIC and Y6. b) CPOM images of PDCBT (left) and PTQ‐10 (right) films printed in the evaporation and LL‐regimes from chloroform. CPOM of images of c) PDCBT and d) PTQ‐10 solutions at 100 mg mL^−1^. (e) *I–V*‐curves of PDCBT:ITIC and PTQ‐10:Y6 systems, and f) device performance comparison between regimes in chloroform. All statistical data are presented as mean ± standard deviation based on 8–12 devices (****p < 0.0001 and *p < 0.05 indicate statistically significant differences between the samples).

To explore the generality of the two assembly pathways accessed by these printing regimes, subsequent complimentary solution and solid‐state characterization was carried out for representative systems for polythiophenes and quinoxalines, two classes of donor polymers that encompass a wide variety of commonly studied systems. The chosen representative material for polythiophenes PDCBT has gained attention recently due to its vast improvement in performance over P3HT when paired with 3,9‐bis(2‐methylene‐(3‐(1,1‐dicyanomethylene)‐indanone))‐5,5,11,11‐tetrakis(4‐hexylphenyl)‐dithieno[2,3‐d:2′,3′‐d’]‐s‐indaceno[1,2‐b:5,6‐b′]dithiophene (ITIC), structural tuneability, and ease of synthesis.^[^ ^70^, ^71^ ^]^ The quinoxaline‐containing polymer system, PTQ‐10, is a top competitor of D18 in terms of balancing low‐cost materials and high performance.^[^ ^72^, ^73^ ^]^ **Figure** **6**c,d shows the CPOM images of both PDCBT and PTQ‐10 solutions at various concentrations as well as their printed films in evaporation and LL regimes. While PDCBT did not show any LC textures in the bulk solution at high concentrations, distinct ordered patterns and point‐defects emerged near the edge of the entrapped solution where the slow edge drying occurred (Figure 6c). Further, PTQ‐10 saw the emergence of schlieren textures in the bulk ≈100mg/mL concentration, as well as dendritically growing nematic LC domains at the drying edge (Figure 6d). Notably, neither PDCBT nor PTQ‐10 appeared to form chiral LC phases as D18 did. This may be due to the solution aggregate structures of these species being less conducive to helical packing. For instance, PDCBT concentration‐dependent SAXS showed contributions from a −2 slope power law, a single semiflexible cylinder corresponding to single polymers, and a pseudo‐Voigt peak. This revealed that a single polymer network persisted despite increasing solution concentration. Similarly, PTQ‐10 concentration‐dependent SAXS showed two semiflexible cylinder contributions corresponding to fibrils and polymers, as well as a pseudo‐Voigt peak. This revealed a persistent fibril aggregate structure (Figure S17, Supporting Information). In both cases, the resiliency of the solution aggregates to undergo changes may lead to limited accessibility to LC‐mesophases, reducing the likelihood of chiral emergence. There may also be some poorly understood physical property of their polymer structures that contribute to the materials’ ability to form chiral hierarchical structures in solution. Regardless, it is clear that, combined with what was seen in the D18 system, the onset of LC mesophases at increasing concentrations is general for a wide range of conjugated donor polymers. Similarly consistent with D18, printing PDCBT and PTQ‐10 films in the evaporation and LL‐regime produced films that were consistently birefringent in the former and isotropic in the latter (Figure 6b). The improved long‐range order in the evaporation regime films corresponds to the onset of LLC mesophases at the meniscus, and the isotropic LL‐regime films are consistent with a random network‐like morphology.

Further, blade‐coated OPV devices were fabricated using the PDCBT and PTQ‐10 systems with the architectures (ITO/PEDOT:PSS/PDCBT:ITIC/PDINN/Ag) and (ITO/PEDOT:PSS/PTQ‐10:Y6/PDINN/Ag) consistent with the D18:Y6 system. The current density‐voltage plots and PCE comparisons are shown in Figure 6e,f. The best performance of PDCBT:ITIC and PTQ‐10:Y6 occurred in the evaporation regime, with average PCE's of 6.7% and 12.0% respectively. On the other hand, the LL‐regime devices demonstrated inferior performances of 4.6% (PDCBT) and 11.2% (PTQ‐10) (Table 1). Directly observing the D18:Y6 PCE results in comparison with these systems (Figure 5b) clearly shows that the trend of improved performances in the evaporation regime is conserved across a diverse set of molecular structures (Figure 6a), demonstrating the possible generality of the LLC assembly pathway and its effect on the consequent devices.

In all three systems studied, the coating regime exerts significant control over the drying dynamics of the active layer, which directly influences the self‐assembly pathways of conjugated polymers. In the evaporation regime, dominance of solvent evaporation creates a pronounced concentration gradient near the triple‐phase contact line. The concentration rise when combined with the capillary flow promotes the formation of LLCs, which enable polymers to assemble into highly ordered and crystalline structures. In contrast, the LL regime leads to the deposition of wet films followed by bulk solvent evaporation, suppressing the formation of LLCs and resulting in less optimal morphologies. As a result, coating the films in the evaporation regime resulted in improved device efficiencies in all three systems with diverse chemical structures.

## 3 Conclusion

In summary, we have reported the crucial role of assembly pathways in dictating device efficiency and stability of OSCs for the first time, using a high‐performing OSC donor polymer D18 and a non‐fullerene acceptor Y6 as a model system and further demonstrated the generality of this finding using other systems including PDCBT:ITIC and PTQ‐10:Y6. Combining microscopy imaging and X‐ray scattering, we demonstrated that the semicrystalline fiber aggregates of D18 first assembles into achiral LC mesophase, which undergoes spontaneous chiral symmetry breaking to form chiral LC mesophase at high solution concentrations. By employing extensive film morphological characterization, we further illustrated that the non‐equilibrium assembly pathway during solution processing depends on the coating regime and the processing solvent to give 1) random fiber aggregation pathway in the LL regime regardless of the solvent and 2) achiral or chiral LC mediated assembly in the evaporation regime depending on the solvent drying time. Compared to the random fiber aggregation pathway, both achiral and chiral assembly pathways improve the film crystallinity by 1.82 and 2.63 times respectively, resulting in 20% and 56% improvements in the PCE as well as 3‐fold and 50‐fold improvements in the T80 lifetime. Our findings further reveal that other conjugated polymers, including PDCBT and PTQ‐10, which undergo LC‐mediated assembly, also exhibit enhanced device efficiencies when processed in the evaporation regime. This underscores the broad applicability and potential of leveraging processing regimes to tune assembly pathways of conjugated polymers for controlling morphology and optimizing device performance.

## 4 Experimental Section

### Materials

The materials used in this work D18 (MW = 74.58 kDa, PDI = 2.38), Y6 (purity > 98%), PTQ‐10 (MW = 62.37 kDa, PDI = 1.76), ITIC (purity >99.0%), PDINN (purity > 98%), and PEDOT:PSS (Al 4083) were purchased from Ossila, Inc. PDCBT (MW = 227.45 kDa, PDI = 5.01) was synthesized at Georgia Institute of Technology. Chlorobenzene (anhydrous, ≥99.8%) and chloroform (≥99.8%) were purchased from Sigma Aldrich. Molecular weights were recorded as weight‐average values. The synthesis details are provided in the Supporting Information.

### Solution Characterizations

SAXS experiments were performed at the 16‐ID beamline of the National Synchrotron Light Source II at Brookhaven National Laboratory to probe the Q ranges between 0.006 < Q < 3 Å^−1^ using multiple Pilatus detectors.^[^ ^74^ ^]^ 10–20 repeated scans were taken on various parts of the sample using X‐ray energy at 13.5 keV with 1 s exposure time to avoid beam damage. Freeze‐drying experiments were conducted to image the structure of aggregates in solution using AFM and SEM. The freeze‐dried samples were prepared by submerging a 0.5 µL solution sandwiched between a Si wafer and a cover glass, in a propane‐ethane mix, followed by storing in liquid nitrogen. The samples stored in liquid nitrogen were then transferred to a Linkam stage where all solvents were sublimated under vacuum for 6–8 h. CPOM measurements of polymer solutions were performed by sandwiching a 1 uL solution between glass coverslips and placing them on a Si wafer to measure using the reflection mode. High‐concentration solutions were prepared via drop and dry method,^[^ ^23^ ^]^ followed by thermally annealing the solutions to 100 °C and cooling back to room temperatures before imaging. The same procedures were followed to prepare the solution samples used for CD measurements. Further details on each characterization technique are described in the Supporting Information.

### Film and Device Characterizations

For OSC fabrication, the conventional architecture (glass/ITO/PEDOT:PSS/D18:Y6/PDINN/Ag, glass/ITO/PEDOT:PSS/PDCBT:ITIC/PDINN/Ag, glass/ITO/PEDOT:PSS/PTQ‐10:Y6/PDINN/Ag) was used. The photoactive layers were prepared by blade coating the blend solutions at varying coating speeds to optimize the device performance in each coating regime. The active layer thickness corresponding to the optimal performance was ≈100 nm. Device performance of OSCs were characterized by using an automated Solar Cell I‐V Test System (Ossila) under AM 1.5G illumination (100 mW cm^−2^) with a class AAA solar simulator from Newport under glovebox‐free or ambient environment, and device stability tests were performed in a glovebox under nitrogen atmosphere without encapsulation at AM 1.5G illumination (100 mW cm^−2^). For film characterization (GIWAXS, PiFM, RSoXS, and c‐AFM), all neat and blend film samples were prepared using the same coating conditions as the optimal OSC devices. GIWAXS measurements were performed at 7.3.3 beamline of the Advanced Light Source at Lawrence Berkeley National Laboratory using incident angles of 0.08°, 0.1°, 0.12°, and 0.14° with X‐ray energy of 10 keV and a beam size of 30 × 50 um. The PiFM measurements were conducted using VistaScope from Molecular Vista, Inc., using Pt coated silicon cantilevers from Molecular Vista. c‐AFM was conducted using the AIST‐NT SmartSPM instrument on the XploRA‐Nano system by Horiba, Inc. BudgetSensors ContGB‐G Au‐coated conductive probes were utilized, with a manufacturer‐specified spring constant of 0.2 N m^−1^ and a probe radius of less than 25 nm. Further details on film and device characterization are available in the Supporting Information.

### Statistical Analysis

The data reported in this work are obtained through necessary normalization and transformation, with the outliers (if any) excluded based on the z‐score test (outliers with z‐score greater than 3 or less than −3 were excluded). All data shown in this work are expressed as mean ± standard deviation (SD). The sample size (n) and calculated *p*‐values are listed in the respective figure legends. The significance between groups was determined using Welch's *t*‐test with alpha = 0.05 (NS: no significance with *p* > 0.05; *p < 0.05; **p < 0.01; ***p < 0.001; ****p < 0.0001). The statistical analysis is carried out using OriginLab.

## Conflict of Interest

The authors declare no conflict of interest.

## Supporting information



Supporting Information

Supplemental Video 1

## Data Availability

The data that support the findings of this study are available from the corresponding author upon reasonable request.

## References

[1] M. Riede , D. Spoltore , K. Leo , Adv. Energy Mater. 2021, 11, 2002653.

[2] S.‐Y. Chang , P. Cheng , G. Li , Y. Yang , Joule 2018, 2, 1039.

[3] V. Pecunia , L. G. Occhipinti , R. L. Z. Hoye , Adv. Energy Mater. 2021, 11, 2100698.

[4] N. C. Davy , M. Sezen‐Edmonds , J. Gao , X. Lin , A. Liu , N. Yao , A. Kahn , Y.‐L. Loo , Nat. Energy 2017, 2, 1.

[5] L. Zhu , M. Zhang , J. Xu , C. Li , J. Yan , G. Zhou , W. Zhong , T. Hao , J. Song , X. Xue , Z. Zhou , R. Zeng , H. Zhu , C.‐C. Chen , R. C. I. MacKenzie , Y. Zou , J. Nelson , Y. Zhang , Y. Sun , F. Liu , Nat. Mater. 2022, 21, 656. 35513501 10.1038/s41563-022-01244-y

[6] Q. Liu , Y. Jiang , K. Jin , J. Qin , J. Xu , W. Li , J. Xiong , J. Liu , Z. Xiao , K. Sun , S. Yang , X. Zhang , L. Ding , Sci. Bull. 2020, 65, 272. 10.1016/j.scib.2020.01.001 36659090

[7] P. Xue , P. Cheng , R. P. S. Han , X. Zhan , Mater. Horiz. 2022, 9, 194. 34679154 10.1039/d1mh01317c

[8] F. Zhao , C. Wang , X. Zhan , Adv. Energy Mater. 2018, 8, 1703147.

[9] S. Park , T. Kim , S. Yoon , C. W. Koh , H. Y. Woo , H. J. Son , Adv. Mater. 2020, 32, 2002217. 10.1002/adma.202002217 33020976

[10] Y. Huang , E. J. Kramer , A. J. Heeger , G. C. Bazan , Chem. Rev. 2014, 114, 7006. 24869423 10.1021/cr400353v

[11] B. Kraabel , C. H. Lee , D. McBranch , D. Moses , N. S. Sariciftci , A. J. Heeger , Chem. Phys. Lett. 1993, 213, 389.

[12] A. J. Heeger , Adv. Mater. 2014, 26, 10.24311015

[13] L. Yang , S. Zhang , C. He , J. Zhang , Y. Yang , J. Zhu , Y. Cui , W. Zhao , H. Zhang , Y. Zhang , Z. Wei , J. Hou , Chem. Mater. 2018, 30, 2129.

[14] J. R. Tumbleston , B. A. Collins , L. Yang , A. C. Stuart , E. Gann , W. Ma , W. You , H. Ade , Nat. Photonics 2014, 8, 385.

[15] Y. Cai , Q. Li , G. Lu , H. S. Ryu , Y. Li , H. Jin , Z. Chen , Z. Tang , G. Lu , X. Hao , H. Y. Woo , C. Zhang , Y. Sun , Nat. Commun. 2022, 13, 2369. 35501300 10.1038/s41467-022-29803-6 PMC9061803

[16] H. W Ro , J. M. Downing , S. Engmann , A. A. Herzing , D. M. DeLongchamp , L. J. Richter , S. Mukherjee , H. Ade , M. Abdelsamie , L. K. Jagadamma , A. Amassian , Y. Liu , H. Yan , Energy Environ. Sci. 2016, 9, 2835. PMC7450673 32863865

[17] L. Zhang , B. Lin , B. Hu , X. Xu , W. Ma , Adv. Mater. 2018, 30, 1800343. 10.1002/adma.201800343 29665119

[18] A. Khasbaatar , A. Cheng , A. L. Jones , J. J. Kwok , S. K. Park , J. K. Komar , O. Lin , N. E. Jackson , Q. Chen , D. M. DeLongchamp , J. R. Reynolds , Y. Diao , Chem. Mater. 2023, 35, 2713.

[19] X. Du , L. Lüer , T. Heumueller , J. Wagner , C. Berger , T. Osterrieder , J. Wortmann , S. Langner , U. Vongsaysy , M. Bertrand , N. Li , T. Stubhan , J. Hauch , C. J. Brabec , Joule 2021, 5, 495.

[20] X. Rodríguez‐Martínez , E. Pascual‐San‐José , M. Campoy‐Quiles , Energy Environ. Sci. 2021, 14, 3301. 34211582 10.1039/d1ee00559f PMC8209551

[21] N. Gyeong An , J. Y Kim , D. Vak , Energy Environ. Sci. 2021, 14, 3438.

[22] A. Khasbaatar , Z. Xu , J.‐H. Lee , G. Campillo‐Alvarado , C. Hwang , B. N. Onusaitis , Y. Diao , Chem. Rev. 2023, 123, 8395. 37273196 10.1021/acs.chemrev.2c00905

[23] K. S. Park , Z. Xue , B. B. Patel , H. An , J. J. Kwok , P. Kafle , Q. Chen , D. Shukla , Y. Diao , Nat. Commun. 2022, 13, 2738. 35585050 10.1038/s41467-022-30420-6 PMC9117306

[24] K. S. Park , X. Luo , J. J. Kwok , A. Khasbaatar , J. Mei , Y. Diao , ACS Cent. Sci. 2023, 9, 2096. 38033802 10.1021/acscentsci.3c00775 PMC10683494

[25] Z. Xu , K. S. Park , J. J. Kwok , O. Lin , B. B. Patel , P. Kafle , D. W. Davies , Q. Chen , Y. Diao , Adv. Mater. 2022, 34, 2203055. 10.1002/adma.202203055 35724384

[26] A. Khasbaatar , A. L. Jones , P. S. Fernando , H. Sai , C. Zhu , E. Gann , J. R. Reynolds , Y. Diao , Chem. Mater. 2024, 36, 2819.

[27] W. L. Tan , L. Tang , R. Matsidik , G. Bryant , T. B. Martin , M. Sommer , D. M. Huang , C. R. McNeill , Macromolecules 2024, 57, 691.

[28] C. Liu , W. Hu , H. Jiang , G. Liu , C. C. Han , H. Sirringhaus , F. Boué , D. Wang , Macromolecules 2020, 53, 8255.

[29] D. Alcazar , F. Wang , T. M. Swager , E. L. Thomas , Macromolecules 2008, 41, 9863.

[30] G. Qu , K. S. Park , P. Kafle , F. Zhang , J. J. Kwok , B. B. Patel , D.‐M. Smilgies , L. Thomsen , C. R. McNeill , Y. Diao , Chem. Mater. 2020, 32, 6043.

[31] C. R. Bridges , M. J. Ford , B. C. Popere , G. C. Bazan , R. A. Segalman , Macromolecules 2016, 49, 7220.

[32] H. Chen , D. Hu , Q. Yang , J. Gao , J. Fu , K. Yang , H. He , S. Chen , Z. Kan , T. Duan , C. Yang , J. Ouyang , Z. Xiao , K. Sun , S. Lu , Joule 2019, 3, 3034.

[33] K. Sun , Z. Xiao , S. Lu , W. Zajaczkowski , W. Pisula , E. Hanssen , J. M. White , R. M. Williamson , J. Subbiah , J. Ouyang , A. B. Holmes , W. W. H. Wong , D. J. Jones , Nat. Commun. 2015, 6, 6013. 25586307 10.1038/ncomms7013 PMC4309440

[34] X. Liao , M. Liu , H. Pei , P. Zhu , X. Xia , Z. Chen , Y. Zhang , Z. Wu , Y. Cui , G. Xu , M. Gao , L. Ye , R. Ma , T. Liu , X. Lu , H. Zhu , Y. Chen , Angew. Chem., Int. Ed. 2024, 63, 202318595. 10.1002/anie.202318595 38224211

[35] Y. Zhao , Z. Huang , X. Kang , J. Yu , M. Ding , D. Liu , G. Lu , X. Bao , L. Yu , M. Sun , Small 2023, 19, 2205244. 10.1002/smll.202205244 36436884

[36] Y. Cui , Y. Xu , H. Yao , P. Bi , L. Hong , J. Zhang , Y. Zu , T. Zhang , J. Qin , J. Ren , Z. Chen , C. He , X. Hao , Z. Wei , J. Hou , Adv. Mater. 2021, 33, 2102420. 10.1002/adma.202102420 34464466

[37] D. Qiu , C. Tian , H. Zhang , J. Zhang , Z. Wei , K. Lu , Adv. Mater. 2024, 36, 2313251. 10.1002/adma.202313251 38702890

[38] Z. Wang , Z. Peng , Z. Xiao , D. Seyitliyev , K. Gundogdu , L. Ding , H. Ade , Adv. Mater. 2020, 32, 2005386. 10.1002/adma.202005386 33150672

[39] F. C. Spano , C. Silva , Annu. Rev. Phys. Chem. 2014, 65, 477. 24423378 10.1146/annurev-physchem-040513-103639

[40] J. J. Kwok , K. S. Park , B. B. Patel , R. Dilmurat , D. Beljonne , X. Zuo , B. Lee , Y. Diao , Macromolecules 2022, 55, 4353.

[41] Y.‐C. Li , K.‐B. Chen , H.‐L. Chen , C.‐S. Hsu , C.‐S. Tsao , J.‐H. Chen , S.‐A. Chen , Langmuir 2006, 22, 11009. 17154578 10.1021/la0612769

[42] Y.‐C. Li , C.‐Y. Chen , Y.‐X. Chang , P.‐Y. Chuang , J.‐H. Chen , H.‐L. Chen , C.‐S. Hsu , V. A. Ivanov , P. G. Khalatur , S.‐A. Chen , Langmuir 2009, 25, 4668. 19366227 10.1021/la803339f

[43] Q. Zhang , W. Wang , S. Zhou , R. Zhang , I. Bischofberger , Nat. Commun. 2024, 15, 7. 38191525 10.1038/s41467-023-43978-6 PMC10774319

[44] D. Chen , M. Nakata , R. Shao , M. R. Tuchband , M. Shuai , U. Baumeister , W. Weissflog , D. M. Walba , M. A. Glaser , J. E. Maclennan , N. A. Clark , Phys. Rev. E 2014, 89, 022506. 10.1103/PhysRevE.89.022506 25353488

[45] V. Borshch , Y.‐K. Kim , J. Xiang , M. Gao , A. Jákli , V. P. Panov , J. K. Vij , C. T. Imrie , M. G. Tamba , G. H. Mehl , O. D. Lavrentovich , Nat. Commun. 2013, 4, 2635. 24189583 10.1038/ncomms3635 PMC3831290

[46] I. Dozov , Europhys. Lett. 2001, 56, 247.

[47] K. S. Park , J. J. Kwok , R. Dilmurat , G. Qu , P. Kafle , X. Luo , S.‐H. Jung , Y. Olivier , J.‐K. Lee , J. Mei , D. Beljonne , Y. Diao , Sci. Adv. 2019, 5, eaaw7757. 31448330 10.1126/sciadv.aaw7757 PMC6688866

[48] Z. Zheng , J. Wang , J. Ren , S. Wang , Y. Wang , W. Ma , L. Zheng , H. Li , Y. Tang , S. Zhang , J. Hou , Sci. Adv. 2023, 9, eadg9021. 37531425 10.1126/sciadv.adg9021 PMC10396288

[49] M. Le Berre , Y. Chen , D. Baigl , Langmuir 2009, 25, 2554. 19437679 10.1021/la803646e

[50] J. M. Ribó , J. Crusats , F. Sagués , J. Claret , R. Rubires , Science 2001, 292, 2063. 11408653 10.1126/science.1060835

[51] K. Swathi , C. Sissa , A. Painelli , K. G. Thomas , Chem. Commun. 2020, 56, 8281. 10.1039/d0cc01922d 32572405

[52] S. Dong , T. Jia , K. Zhang , J. Jing , F. Huang , Joule 2004, 2020, 4.

[53] Y. Fu , T. H. Lee , Y.‐C. Chin , R. A. Pacalaj , C. Labanti , S. Y. Park , Y. Dong , H. W. Cho , J. Y. Kim , D. Minami , J. R. Durrant , J.‐S. Kim , Nat. Commun. 2023, 14, 1870. 37015916 10.1038/s41467-023-37234-0 PMC10073232

[54] J. Rivnay , S. C. B. Mannsfeld , C. E. Miller , A. Salleo , M. F. Toney , Chem. Rev. 2012, 112, 5488. 22877516 10.1021/cr3001109

[55] T. Liu , H.‐K. Chan , D. Wan , Soft Matter 2023, 19, 7313. 37697926 10.1039/d3sm00680h

[56] K. Olsen , J. Bohr , Theor. Chem. Acc. 2010, 125, 207.

[57] P. Pierański , Comput. Methods Sci. Technol. 1998, 4, 9.

[58] X. Dong , Y. Jiang , L. Sun , F. Qin , X. Zhou , X. Lu , W. Wang , Y. Zhou , Adv. Funct. Mater. 2022, 32, 2110209.

[59] G. Qu , J. J. Kwok , Y. Diao , Acc. Chem. Res. 2016, 49, 2756. 27993010 10.1021/acs.accounts.6b00445

[60] R. D. Deegan , O. Bakajin , T. F. Dupont , G. Huber , S. R. Nagel , T. A. Witten , Nature 1997, 389, 827. 10.1103/physreve.62.756 11088531

[61] S. Mo Park , D. Ki Yoon , Mater. Horiz. 2024, 11, 1843. 38375871 10.1039/d3mh01585h

[62] J. H. Carpenter , M. Ghasemi , E. Gann , I. Angunawela , S. J. Stuard , J. J. Rech , E. Ritchie , B. T. O'Connor , J. Atkin , W. You , D. M. DeLongchamp , H. Ade , Adv. Funct. Mater. 2018, 29, 201806977. PMC7552815 33061870

[63] J. Jahng , D. A. Fishman , S. Park , D. B. Nowak , W. A. Morrison , H. K. Wickramasinghe , E. O. Potma , Acc. Chem. Res. 2015, 48, 2671. 26449563 10.1021/acs.accounts.5b00327

[64] D. Nowak , W. Morrison , H. K. Wickramasinghe , J. Jahng , E. Potma , L. Wan , R. Ruiz , T. R. Albrecht , K. Schmidt , J. Frommer , D. P. Sanders , S. Park , Sci. Adv 2016, 2, e1501571. 27051870 10.1126/sciadv.1501571 PMC4820382

[65] B. Qiu , L. Xue , Y. Yang , H. Bin , Y. Zhang , C. Zhang , M. Xiao , K. Park , W. Morrison , Z.‐G. Zhang , Y. Li , Chem. Mater. 2017, 29, 7543.

[66] Z. Chen , X. Chen , B. Qiu , G. Zhou , Z. Jia , W. Tao , Y. Li , Y. M. Yang , H. Zhu , J. Phys. Chem. Lett. 2020, 11, 3226. 32259443 10.1021/acs.jpclett.0c00919

[67] J. Song , L. Ye , C. Li , J. Xu , S. Chandrabose , K. Weng , Y. Cai , Y. Xie , P. O'Reilly , K. Chen , J. Zhou , Y. Zhou , J. M. Hodgkiss , F. Liu , Y. Sun , Adv. Sci. 2020, 7, 2001986. 10.1002/advs.202001986 PMC7509652 32999853

[68] Y. W. Soon , S. Shoaee , R. S. Ashraf , H. Bronstein , B. C. Schroeder , W. Zhang , Z. Fei , M. Heeney , I. McCulloch , J. R. Durrant , Adv. Funct. Mater. 2014, 24, 1474.

[69] P. S. Fernando , J. M. Mativetsky , ACS Appl. Energy Mater. 2023, 6, 10951.

[70] Q. Wang , M. Li , X. Zhang , Y. Qin , J. Wang , J. Zhang , J. Hou , R. A. J. Janssen , Y. Geng , Macromolecules 2019, 52, 4464.

[71] Y. Qin , M. A. Uddin , Y. Chen , B. Jang , K. Zhao , Z. Zheng , R. Yu , T. J. Shin , H. Y. Woo , J. Hou , Adv. Mater. 2016, 28, 9416. 27600932 10.1002/adma.201601803

[72] C. Sun , F. Pan , S. Chen , R. Wang , R. Sun , Z. Shang , B. Qiu , J. Min , M. Lv , L. Meng , C. Zhang , M. Xiao , C. Yang , Y. Li , Adv. Mater. 2019, 31, 1905480. 10.1002/adma.201905480 31867848

[73] Y. Wu , Y. Zheng , H. Yang , C. Sun , Y. Dong , C. Cui , H. Yan , Y. Li , Sci. China Chem. 2020, 63, 265.

[74] L. Yang , J. Liu , S. Chodankar , S. Antonelli , J. DiFabio , J. Synchrotron Radiat. 2022, 29, 540. 35254319 10.1107/S1600577521013266 PMC8900859

