# Supplementary material for: Lyotropic Liquid Crystal Mediated Assembly of Donor Polymers Enhances Efficiency and Stability of Blade‐Coated Organic Solar Cells
Source: Adv Mater. 2025 Feb 5;37(11):2414632. doi: 10.1002/adma.202414632 (PMC11923519; doi:10.1002/adma.202414632)
Supplement: Supplementary file 1 — Supporting Information [file ADMA-37-2414632-s002.docx]

**Supporting Information for**

**Lyotropic Liquid Crystal Mediated Assembly of Donor Polymers Enhance Efficiency and Stability of Blade-Coated Organic Solar Cells**

*Azzaya Khasbaatar*†*,^1^ Alec M. Damron*†*,^1^ Pravini S. Fernando,^1^ Jasmine S. Williams,^1^ Chenhui Zhu,^2^ Eliot Gann,^3^ Jong-Hoon Lee,^4^ Adrian Birge^5^, Bora Kim^6^, Sina Sabury^7^, Minjoo L. Lee^6^, John Reynolds^7^, and Ying Diao^1,8*^*

^1^ Department of Chemical and Biomolecular Engineering, University of Illinois at Urbana-Champaign, 600 South Mathews Avenue, Urbana, Illinois 61801, USA

^2^ Advanced Light Source, Lawrence Berkeley National Laboratory, Berkeley, California 94720, USA

^3^ Materials Measurement Laboratory, National Institute of Standards and Technology, Gaithersburg, Maryland 20899, USA

^4^ Department of Advanced Materials Engineering, Kyonggi University, Suwon 16227, Republic of Korea

^5^ Department of Materials Science and Engineering, University of Illinois at Urbana-Champaign 1304 W. Green St., Urbana, IL 61801, USA

^6^ Department of Electrical and Computer Engineering, University of Illinois at Urbana-Champaign, 306 N. Wright St., Urbana, IL 61801, USA

^7^ School of Chemistry and Biochemistry, Georgia Institute of Technology, North Avenue
Atlanta, GA 30332, USA

^8^ Beckman Institute, University of Illinois at Urbana-Champaign, Urbana, Illinois 61801, USA

†Contributed equally, co-first author.

*For correspondence: [yingdiao@illinois.edu](mailto:yingdiao@illinois.edu)

## **Experimental Methods**

***PDCBT synthesis procedure***

PDCBT is synthesized as shown below in Figure S1. Intermediate molecules 1, 2, 3, and 4 were synthesized following the reported procedures^[1]^.


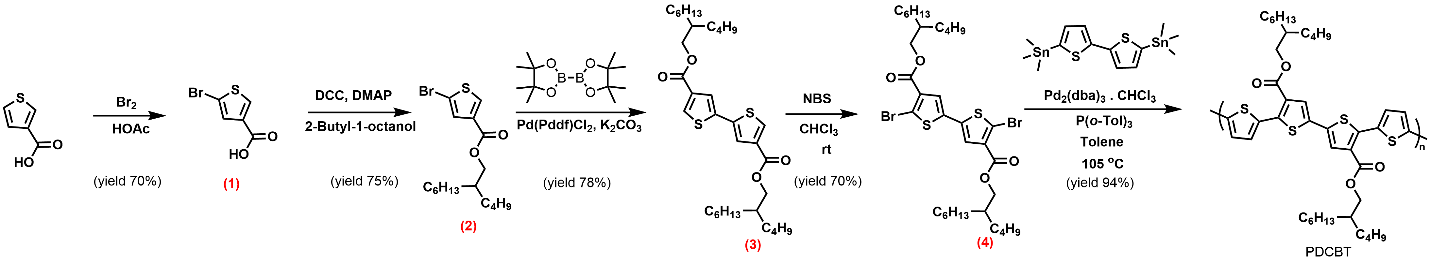


**Figure S1.** Synthesis pathway for PDCBT. yields are reported in parenthesis.

***polymerization and purification procedure***

Dibromo bithiophene comonomer (molecule 4) (236 mg, 0.315 mmol, 1 equiv.) and 5,5'-bis(trimethylstannyl)-2,2'-bithiophene (155 mg, 0.315 mmol, 1 equiv.) were added to a freshly dried and cooled round bottom flask containing a stir bar. Then the flask was taken to a glove box where dipalladiumtris(dibenzylideneacetone)chloroform complex, Pd2(dba)3·CHCl3, as the catalyst (9.9 mg, 0.00945 mmol, 0.03 equiv.), tris(o-tolyl)phosphine as the ligand, and 4.5 mL of toluene as the polymerization solvent were added to the flask. The reaction flask then was taken out of the glove box and was immersed in an oil bath set at 105 °C and stirred for 16 hours. At the end of this time, a small amount of palladium scavenger (diethylammonium diethyldithiocarbamate) was added, and the temperature dropped to 90 °C. After stirring for 1 hour at 90 °C, the solution was brought to room temperature, and the crude polymer was precipitated into stirring cold methanol upon cooling to room temperature. The crude polymer was further purified by Soxhlet washing with methanol (24 hours), Acetone (24 hours), hexane (24 hours), and the purified polymer was obtained from Soxhlet extraction using chloroform. The purified polymer then reprecipitated into cold methanol and collected by vacuum filtration on a nylon membrane with pore size of 45 μm.

PDCBT: Dark-red solid (223 mg, 94% yield). Number-average molecular weight: 45.40 kDa, weight-average molecular weight: 227.06 kDa, dispersity (Đ): 5.01. (GPC in 140 °C 1,2, 4-trichlorobenzene vs polystyrene). ^1^H NMR (700 MHz, CHCl_3_, 25 °C): δ(ppm) 7.56 (s, 2H), 7.48 (s, 2H), 4.23 (d, J = 5.25 Hz, 4H), 1.77 (m, 2H), 1.32-1.25 (m, 32H), 0.91-0.86 (m, 12H).


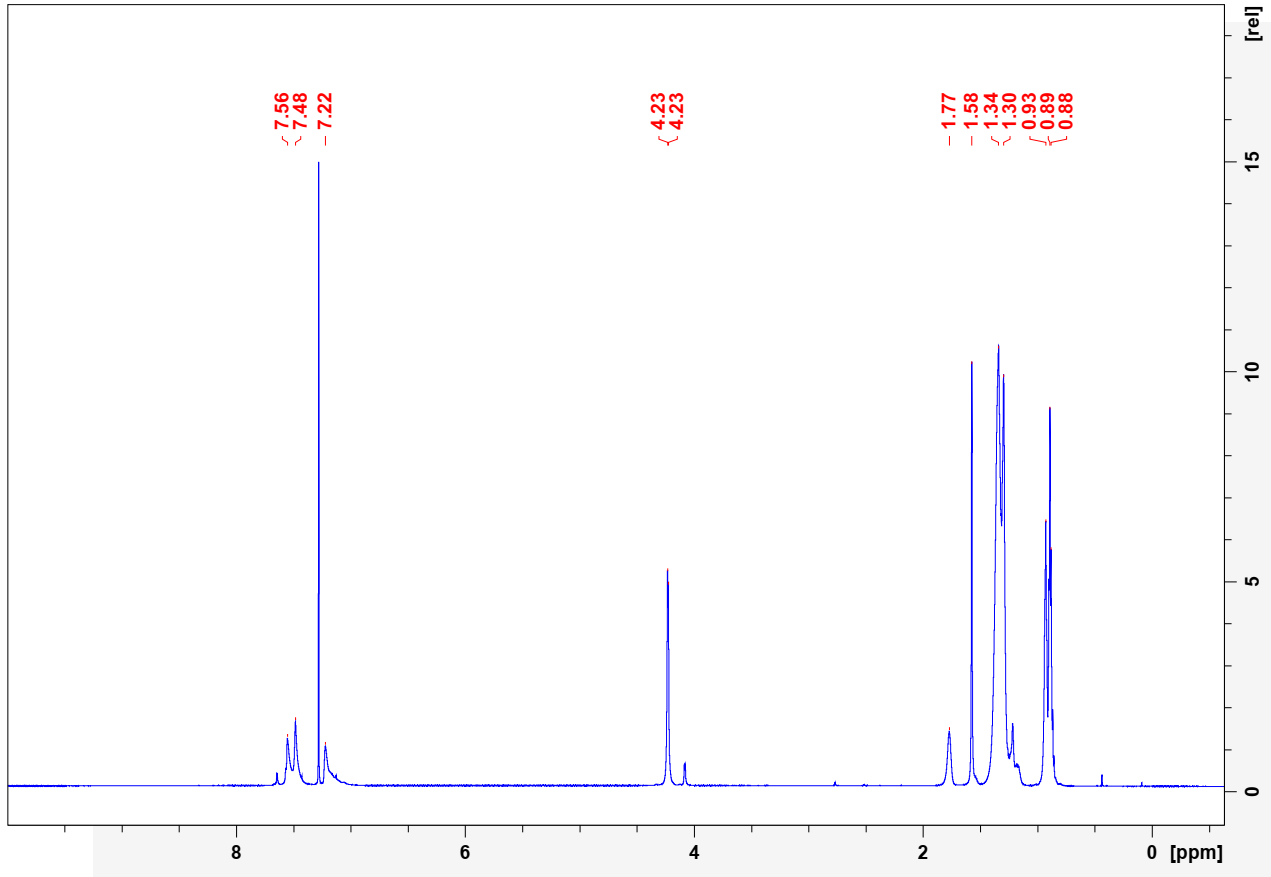


**Figure S2.** ^1^H NMR of PDCBT in CDCl_3_ at 25 °C.


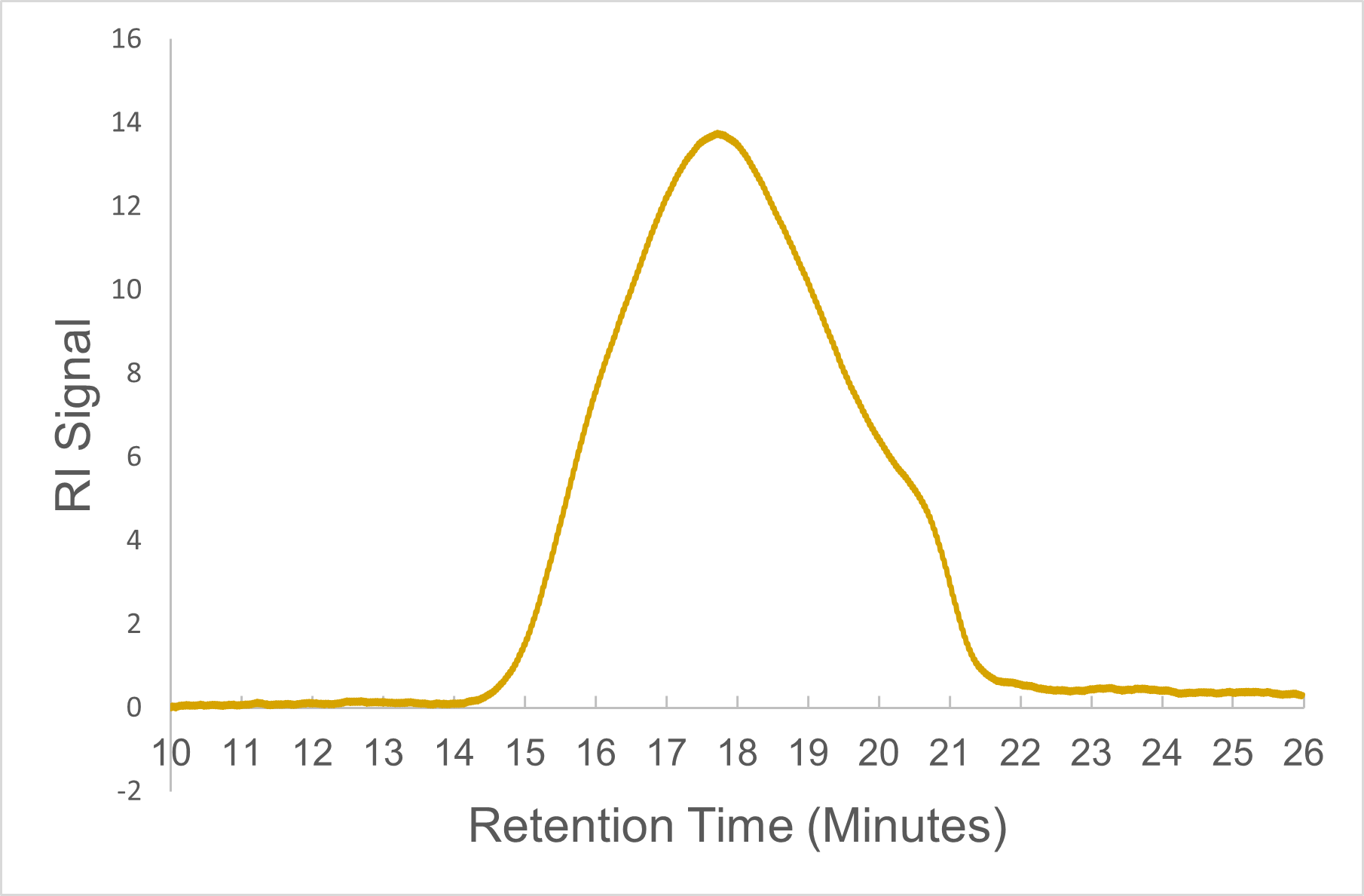


**Figure S3.** High-temperature GPC trace of PDCBT in trichlorobenzene at 140 °C.

***UV–Vis Spectroscopy***

UV–vis (Cary 60 UV–Vis, Agilent) spectroscopy was used to measure the absorbance of 5 mg/mL solutions of D18 and 8 mg/mL solutions of Y6 in chlorobenzene and chloroform solvents in a short path length of 10 μm quartz cell (Starna Cells). Before each solution measurement, a blank solvent measurement for each solvent was taken for background subtraction.

***Solution characterization using SAXS***

SAXS experiments were performed at the 16-ID beamline of the National Synchrotron Light Source II at Brookhaven National Laboratory to probe the Q ranges between 0.006 < Q < 3 Å^-1^ using multiple Pilatus detectors.^[2]^ For SAXS experiment, neat polymer samples were dissolved in chlorobenzene, stirred at 40 °C overnight, and brought to room temperature at least 2-3 before the measurement. The samples were measured using a 1.5 mm diameter quartz capillary. Due to the high viscosity of the 50 mg/mL solution, it was thermally annealed to 85 °C to be able to transfer to the measurement capillary and then brought to room temperature for at least 30 minutes before the measurement. During the measurement, 10-20 repeated scans were taken on various parts of the sample using X-ray energy at 13.5 keV with 1 s exposure time to avoid beam damage. The scattering profiles were then obtained by averaging the repeated measurements and subtracting the empty cell and blank solvent contributions using a Python package *py4xs* in Jupyter notebooks.^[3]^ The model fitting of the scattering profiles was performed using SASView program with dI data for weighting, Levenberg–Marquardt with 200 steps for the algorithm where the uncertainties are determined from the covariance matrix, and χ^2^ values and the residuals plot were used as the goodness of fit metrics.

***Freeze-Drying Method for Imaging the Solution-State Aggregates***

Freeze-drying experiments were conducted to image the structure of aggregates in solution using AFM and SEM. A 0.5 uL solution at the desired solution concentration was first sandwiched in between a Si wafer and a thin microscope cover glass (VWR Micro cover glasses). The solution was then submerged in a liquid mixture of 63% propane and 37% ethane for about a minute and then stored in liquid nitrogen. The microscope glass slide was then removed in the liquid nitrogen bath, followed by quickly transferring the sample on the Si wafer to a Linkam stage at −100 °C under vacuum. The Linkam stage temperature is then slowly increased at a rate of 0.5 °C/min to −80 °C (far below the melting points of chlorobenzene at mp = −45 °C and chloroform mp = -63 °C) followed by 6–8 h of constant monitoring with cross-polarized microscopy to ensure sublimation of all solvent. The freeze-dried samples were then imaged by the Cypher AFM from Asylum Research using tapping mode and SEM (JEOL 7000F) at 3-5 keV accelerating voltage.

***Device Fabrication***

OSCs were fabricated using the conventional architecture (glass/ITO/PEDOT:PSS/D18: Y6/PDINN/Ag, glass/ITO/PEDOT:PSS/PDCBT:ITIC/PDINN/Ag, glass/ITO/PEDOT:PSS/PTQ-10:Y6/PDINN/Ag). Before film deposition, 20 × 15 cm ITO-patterned glass substrates (Ossila, Inc.) were cleaned with toluene, acetone, and isopropyl alcohol. The cleaned substrates were then dried with nitrogen and plasma treated (Harrick Plasma PDC-001-HP) for 6 min at 300 mT of dry air and high power (30 W). To deposit the hole transporting layer, PEDOT:PSS solution was filtered with a 0.45 μm PTFE syringe filter before deposition. The filtered solution was deposited on the cleaned ITO substrates by spin-coating for 30 s at 4000 rpm in an ambient atmosphere, which resulted in an active layer thickness of ≈ 20–30 nm measured by a Bruker Dektak XT profilometer. After spin-coating, the PEDOT:PSS layer was annealed at 150 °C for 20 min followed by slow cooling to room temperature for active layer deposition. The photoactive layer solution for all devices was prepared by dissolving D18 and Y6 at 1:1.6 (wt/wt) ratio in CB and CF at a total concentration of 13 mg/mL, PDCBT and ITIC at 1:1 ratio in CF at a total concentration of 10 mg/mL, and PTQ-10 and Y6 at 1:1.2 ratio in CF at a concentration of 15.4 mg/mL without any additives. The resulting blend solution was stirred at 40 °C overnight and brought to room temperature and stirred for 2-3 hours before deposition by blade coating. The blend solutions were then blade coated using a blade angle of 25° using a solution volume of 3 uL for CB with coating speeds varied from 0.03 to 50 mm/s and 5 uL for CF solutions with coating speeds varied from 0.2 to 100 mm/s. The active layer thickness corresponding to the optimal coating conditions was ~100 nm. After blade coating, 1 mg/mL PDINN solution in methanol was spin coated at 3000 rpm for 30 seconds, followed by Ag electrode deposition using Thermal Evaporator System from Angstrom Engineering.

***Device Characterization***

The current density (*J*–*V*) curves were characterized by using an automated Solar Cell I-V Test System (Ossila) under AM 1.5G illumination (100 mW/cm^2^) with a class AAA solar simulator from Newport under glovebox-free or ambient environment. Before each test, the solar simulator was calibrated by reference single-crystal Si cells (Sciencetech). A total of 8-12 devices were tested under each condition. The active area of all devices is 0.04 cm^2^. For calculating the device performance, mismatch factor was not applied. Device stability tests were performed in a glovebox under nitrogen atmosphere without encapsulation at AM 1.5G illumination (100 mW/cm^2^).

***Solution characterization using CPOM***

Solution characterization under CPOM was performed by sandwiching the polymer solutions in between two microscope glass coverslips placed on a Si wafer during the CPOM measurements using the reflection mode. High concentration solutions were obtained by the drop and dry method,^[4]^ where the concentration of the pristine solution is increased by drying a certain amount of solution and then adding a 1 uL of solution to the dried droplet and mixing the solution by shearing with the microscope cover glasses to increase the concentration. For instance, 200 mg/mL solution was achieved by drying 9 uL of 20 mg/mL solution and adding 1 uL solution to increase the concentration of 20 mg/mL solution by ten-fold. The solution samples then underwent thermal annealing to 100 °C reach the isotropic phase and then cooled down to room temperature at 10 °C/min (slow cooling) or rapidly quenched to room temperature before imaging under CPOM.

***CD measurements***

CD spectra were recorded by using JASCO J-1500 spectrophotometer. For all solution and solid samples, the background contributions were subtracted. To eliminate contributions from linear dichroism, CD spectra were measured at numerous sample rotation angles to obtain the average.

***GIWAXS***

GIWAXS measurements were performed at 7.3.3 beamline of the Advanced Light Source at Lawrence Berkeley National Laboratory using incident angles of 0.08°, 0.1°, 0.12°, and 0.14° with X-ray energy of 10 keV and a beam size of 30 x 50 um. All samples were placed in a helium chamber for measurement. Both neat and blend GIWAXS samples were prepared on Si wafer the substrate using the same blade coating conditions as the optimal OSC devices were fabricated. From GIWAXS measurement, 2D scattering images were obtained and converted to 1D linecut profiles by using the beamline NIKA package in Igor Pro.^[5]^ The Ewald sphere correction (missing wedge correction) for the 2D images were obtained using the WAXSTools plugin^[6]^ in Igor Pro. The intensity of (010) peak at each χ angle was determined by gaussian peak deconvolution analysis. For rDoC analysis, geometrically corrected intensities of (002) peak from 5° sector cut profiles within the range of 10° < χ < 80° were integrated and normalized by the total illumination volume (thickness and area of the sample).

***RSoXS***

RSoXS data were collected at the National Synchrotron Light Source II at the beamline 7-ID-1 in the transmission geometry. For sample preparation, the photoactive layer was blade coated and thermally annealed using the same device fabrication conditions on a polystyrene sulfonate (PSS) film, which was spin coated from a 10% PSS solution in water. The PSS solution was stirred overnight at room temperature before depositing on a Si wafer substrate. After photoactive layer deposition, the sample on a Si substrate was immersed in water, which dissolved the PSS layer and separated the sample from the substrate. The sample was then picked up with a 1 × 1 mm, 100 nm-thick Si_3_N_4_ membrane supported on a 5 × 5 mm, 200 μm-thick Si frame (Norcada) and transferred into the vacuum chamber for RSoXS measurement. RSoXS measurements were performed at the beam energy from 270 to 290 eV with 5 s of exposure time per scan. The collected 2D scattering images were then converted into 1D profiles using the Nika package supported in the Igor Pro environment.

***PiFM measurements***

The PiFM measurements were conducted using VistaScope from Molecular Vista, Inc., using Pt coated silicon cantilevers from Molecular Vista. Before starting the measurement, the laser power was recorded within the IR range of 733 – 1960 cm^-1^, followed by the laser alignment using a parabolic mirror to ensure that the laser was focused on the tip of the cantilever. The wavelength of the quantum cascade laser (QCL) used for alignment was tuned to 1750 cm^-1^ at an intensity of 20 % of the recorded maximum power. For PiFM imaging, a scan size of 1 μm x 1 μm with a pixel size of 256 x 256 was used at a speed of 1 line/sec. During the measurement, 1437 and 823 cm^-1^ laser was tuned to <5% of the maximum power with 30 seconds duration per spectrum. To obtain PiFM spectra of the neat films, QCL laser was first turned off to obtain only AFM topography images using a scan size of 1 μm x 1 μm. Afterwards, point spectra were obtained based on the collected AFM topography images with an IR intensity of <5 % of the maximum power with 20-30 s duration per spectrum. The bulk FTIR spectra of neat materials using solid powder samples were also obtained using Thermo Nicolet iS50 spectrometer as a comparison with the PiFM spectra on neat films.

***c-AFM measurements***

Hole current maps were acquired using the AIST-NT SmartSPM instrument on the XploRA-Nano system by Horiba, Inc. BudgetSensors ContGB-G Au-coated conductive probes were utilized, with a manufacturer-specified spring constant of 0.2 N/m and a probe radius of less than 25 nm. A substrate bias voltage of +3 V was applied, and the probe-sample force ranged from 5-7 nN. To ensure hole-only transport, high work function top (Au probe) and bottom electrodes (PEDOT:PSS coated ITO glass) were used.

***Film Thickness Measurements***

Film thickness was measured by using the Bruker Dektak XT profilometry with 12.5 um radius stylus. During thickness measurements, a stylus force of 3 mg and a scan range of 1000–2000 μm were used at a speed of 50 μm/s. For thickness measurements, a part of the film was removed with a cotton swab with toluene. The thickness measurements were repeated in three to five different positions to obtain the average and standard deviation.

**Figure S4**. SAXS deconvolution of the model fit using the double flexible cylinder model.

**Table S1.** Model fitting results for 50 mg/mL solution of D18 in CB.

| **Parameters** | **Values** |
| --- | --- |
| *L_1_* (nm) | 100* |
| *L_p1_* (nm) | >>*R_1_* |
| *R_1_* (Å) | 6.2 ± 0.3 |
| *q_1_* (Å^-1^) | 0.218 ± 0.002 |
| *FWHM_1_* | 0.258 ± 0.008 |
| *w_1_* | 0 |
| *q_2_* (Å^-1^) | 0.554 ± 0.001 |
| *FWHM_2_* | 0.019 ± 0.002 |
| *L_2_* (nm) | 100* |
| *L_p2_* (nm) | 46.1 ± 10.5 |
| *R_2_* (nm) | 6.3 ± 0.2 |
| *Polydispersity* | 0.28 |

**Description of the fitting parameters:**

| **Parameters** | **Description** |
| --- | --- |
| *L_1_* | Length of flexible cylinder 1, corresponding to the length of polymer chains |
| *L_p1_* | Persistence length of flexible cylinder 1, corresponding to the persistence length of polymer chains |
| *R_1_* | Radius of flexible cylinder 1, corresponding to the radius of polymer chains |
| *q_1_* | Location of the pseudo-Voigt peak |
| *FWHM_1_* | Full width half maximum of the pseudo-Voigt peak |
| *w_1_* | Lorentzian fraction of the pseudo-Voigt peak |
| *q_2_* | Location of the Lorentz peak |
| *FWHM_2_* | Full width half maximum of the Lorentz peak |
| *L_2_* | Length of flexible cylinder 2, corresponding to the length of fibers |
| *L_p2_* | Persistence length of flexible cylinder 2, corresponding to the persistence length of fibers |
| *R_2_* | Radius of flexible cylinder 2, corresponding to the radius of fibers |
| *Polydispersity* | Polydispersity in the radius of fibers |

**Figure S5.** Temperature dependent SAXS measurements for 50 mg/mL solution of D18 in chlorobenzene, suggesting that the intensity of the fiber aggregates decreases with increasing temperature.


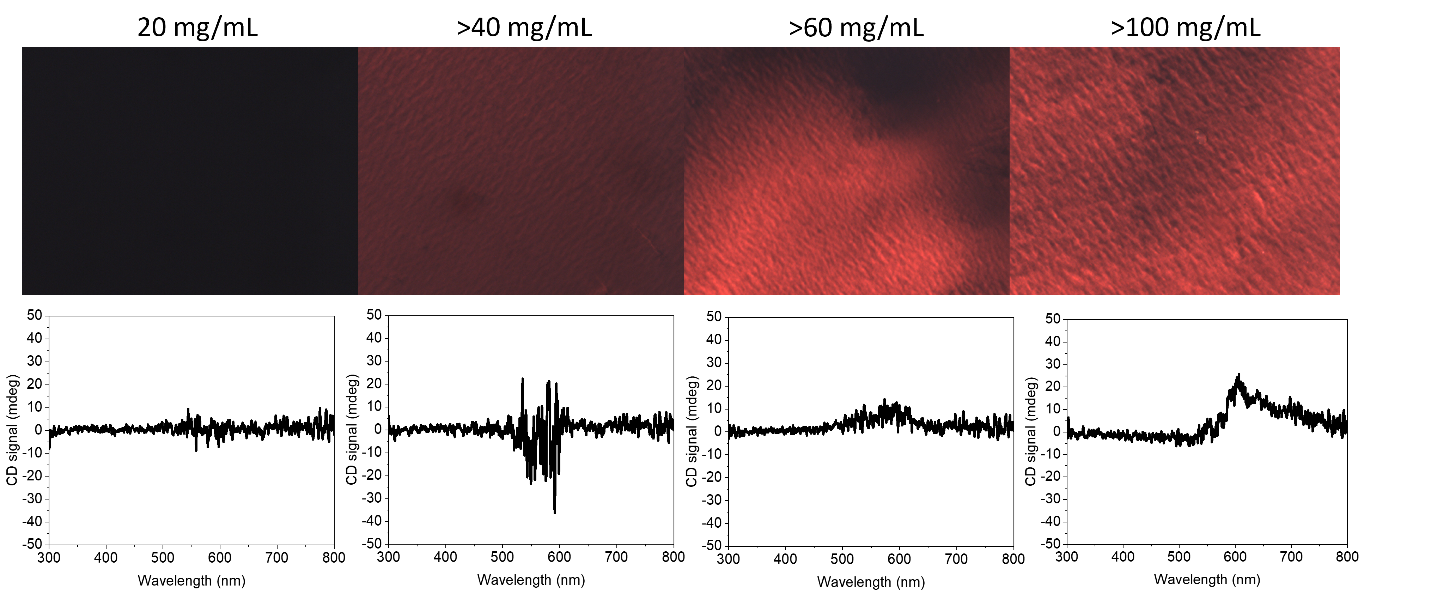


**Figure S6.** CPOM images of D18 CF solutions at different concentrations. The solutions were prepared by heating the solution to 55 °C and cooling down to room temperature at a cooling rate of 10 °C/min.


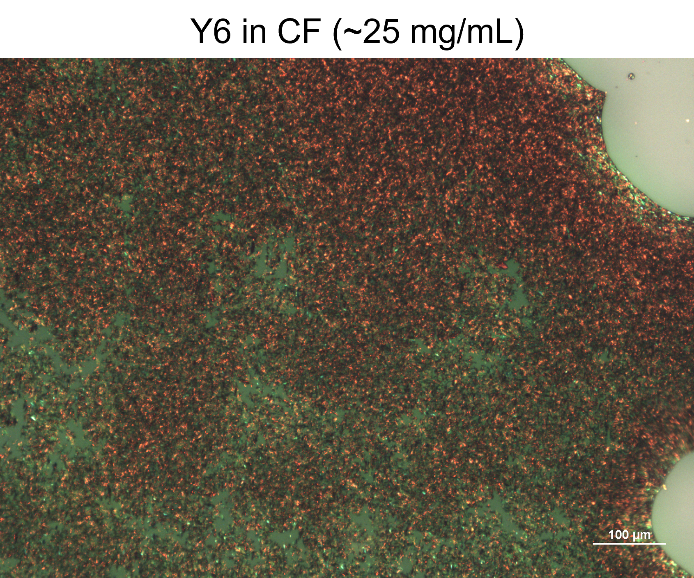


**Figure S7.** CPOM image of ~ 25 mg/mL solution of Y6 in CF showing formation of large crystals upon reaching its solubility limit


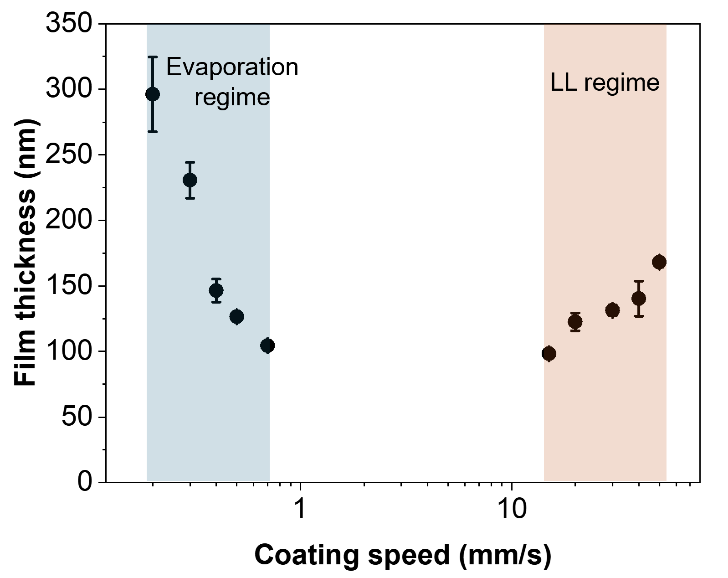


**Figure S8.** Film thickness versus coating speed relationship plot for D18:Y6 films processed from CF as the main solvent. Other relevant blade coating conditions: blade angle = 25°, solution volume = 8 uL, blade width = 6 mm. The film thicknesses within the transition regime were not obtained since the thickness is too low that it is irrelevant for device fabrication.


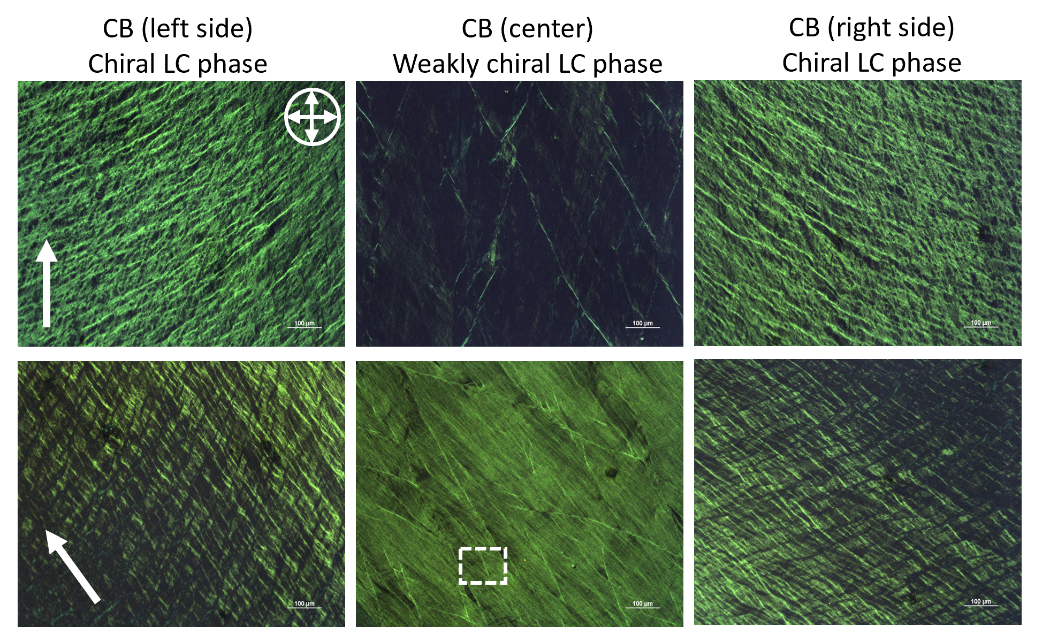


**Figure S9.** CPOM images of the left and right edge of the D18:Y6 CB film coated in the evaporation regime. The film coating direction is along the arrows indicated.


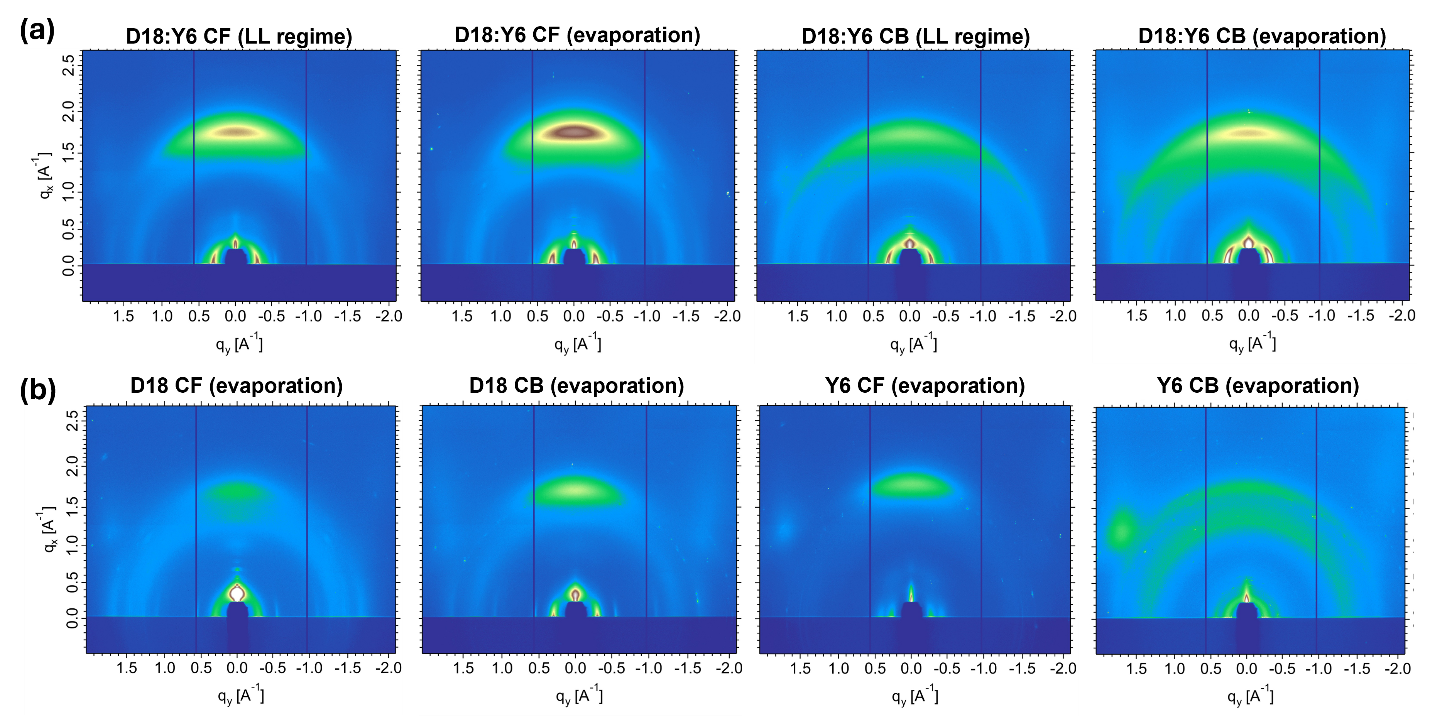


**Figure S10**. 2D GIWAXS patterns of (a) D18:Y6 blend films coated from CB and CF in the evaporation and LL regimes and (b) D18 and Y6 neat films coated from CB and CF in the evaporation regime.


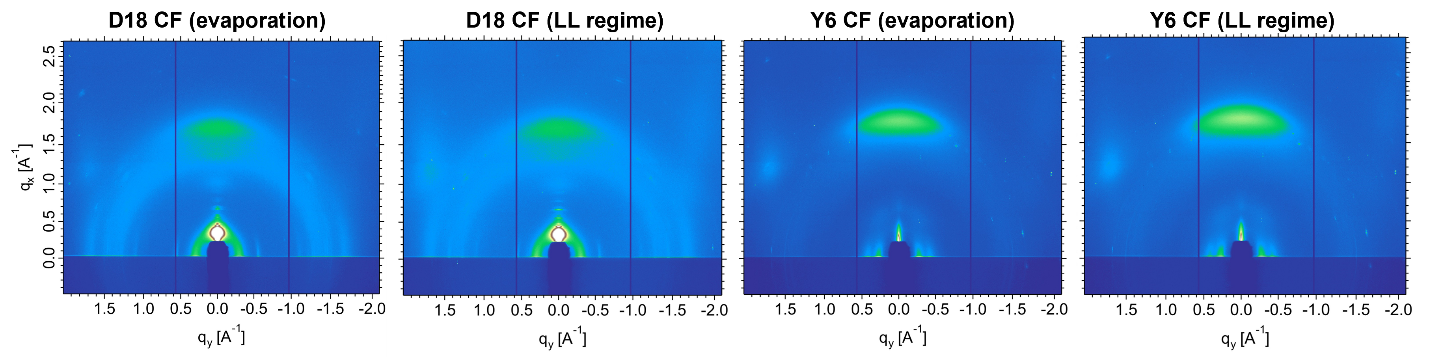


**Figure S11.** 2D GIWAXS patterns and D18 and Y6 neat films coated from CF in the evaporation and LL regimes.


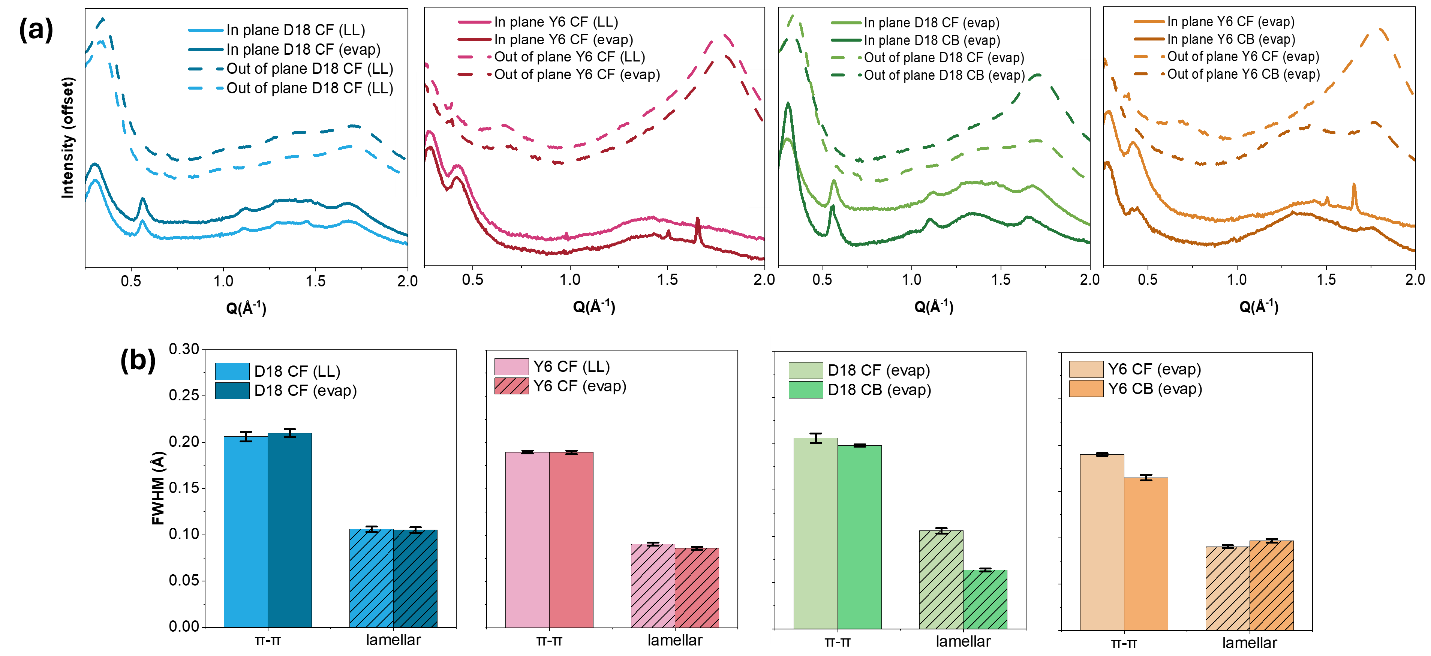


**Figure S12.** (a) In-plane and out-of-plane sector profiles of the neat films (D18 and Y6) comparing the effect of regimes and solvents. (b) Comparison of the full width half maximum (FWHM) of the π-π stacking and lamellar stacking peaks based on the 1D profiles in (a).

**Table S2.** FWHM and peak positions of (010) and (100) peaks of the neat films.

| **Samples** | **(100) position (Å^-1^)** | **FWHM of (100) peak (Å^-1^)** | **(010) position (Å^-1^)** | **FWHM of (010) peak (Å^-1^)** |
| --- | --- | --- | --- | --- |
| D18 CF (evap) | 0.30 | 0.106 ± 0.003 | 1.74 | 0.206 ± 0.005 |
| D18 CF (LL) | 0.30 | 0.105 ± 0.003 | 1.72 | 0.210 ± 0.004 |
| Y6 CF (evap) | 0.27 | 0.086 ± 0.001 | 1.79 | 0.189 ± 0.002 |
| Y6 CF (LL) | 0.27 | 0.090 ± 0.002 | 1.78 | 0.189 ± 0.001 |
| D18 CB (evap) | 0.31 | 0.063 ± 0.002 | 1.71 | 0.198 ± 0.001 |
| Y6 CB (evap) | 0.27 | 0.097 ± 0.002 | 1.78 | 0.165 ± 0.003 |


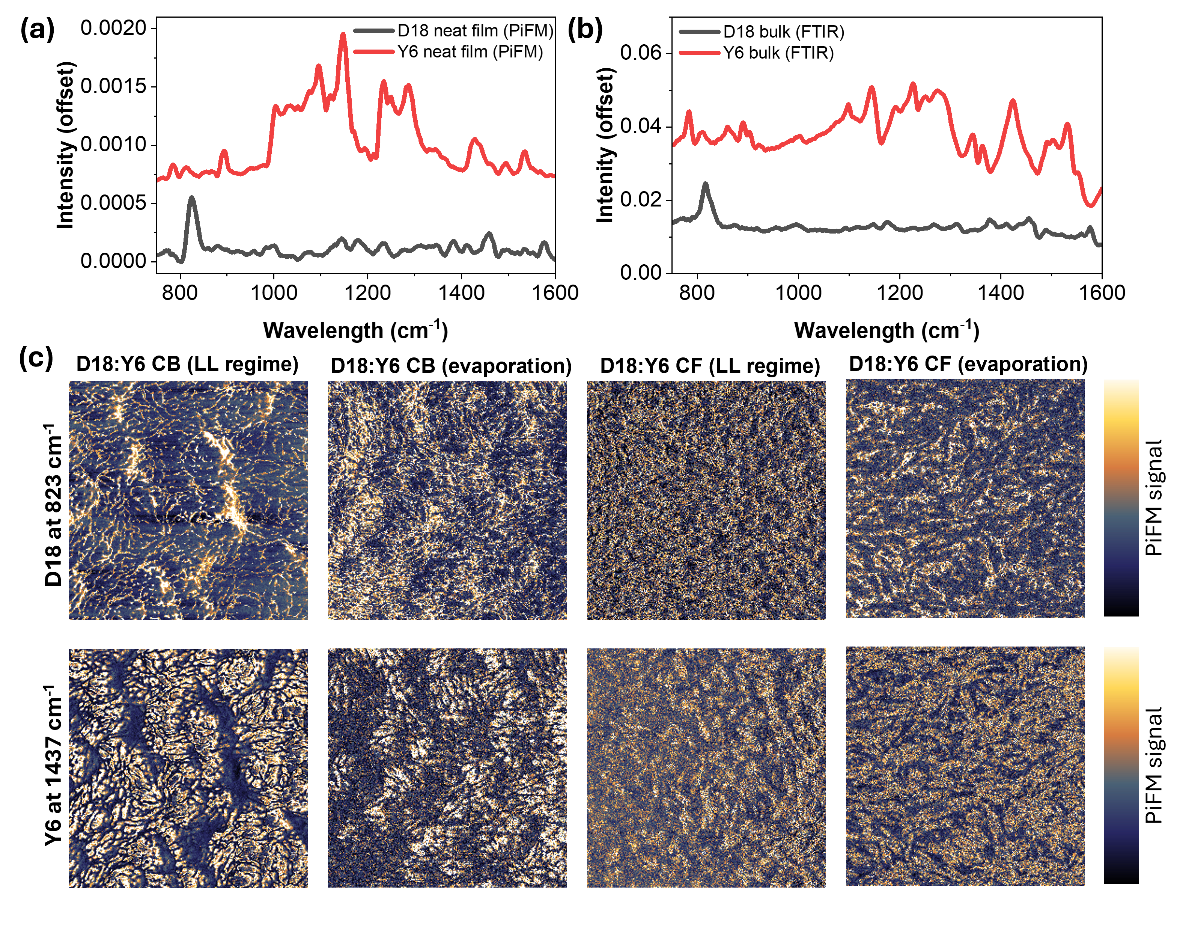


**Figure S13.** (a) PiFM spectra of the neat films of D18 and Y6 coated in the evaporation regime. (b) FTIR spectra of D18 and Y6 powders. (c) PiFM measurements of the blend films probed at 823 cm^-1^, showing the D18 domains (top) and at 1437 cm^-1^, showing the Y6 domains (bottom).


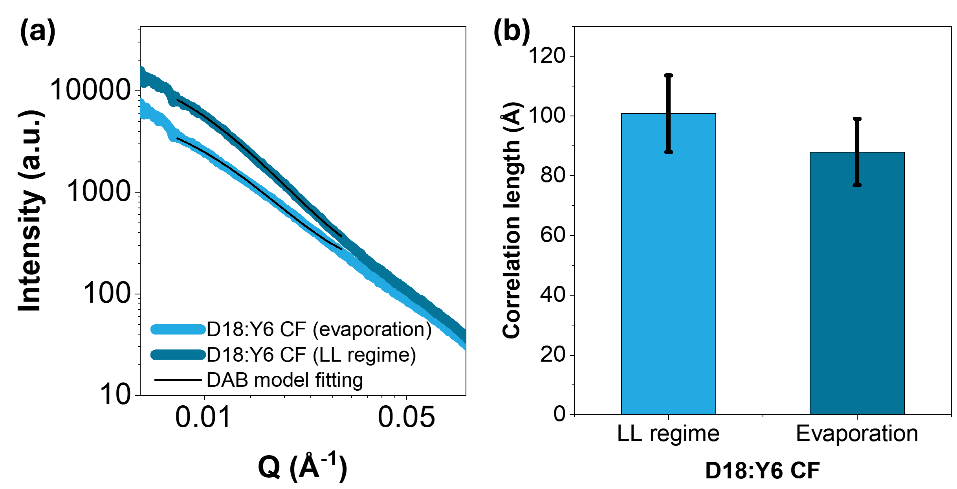


**Figure S14.** (a) Resonant soft X-ray scattering (RSoXS) profiles of D18:Y6 CF blend films depending on the coating regimes. (b) Correlation length comparison based on fitting the RSoXS profiles to the Debye-Anderson-Brumbereger (DAB) model, showing no differences in the domain spacing.


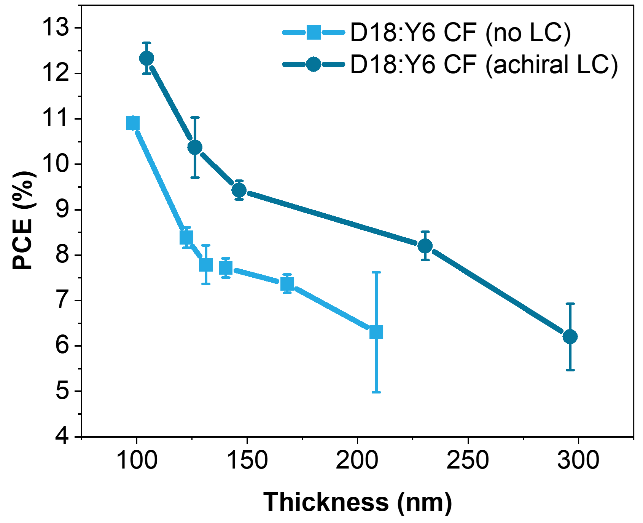


**Figure S15.** Power conversion efficiencies as a function of thickness for D18:Y6 CF devices.


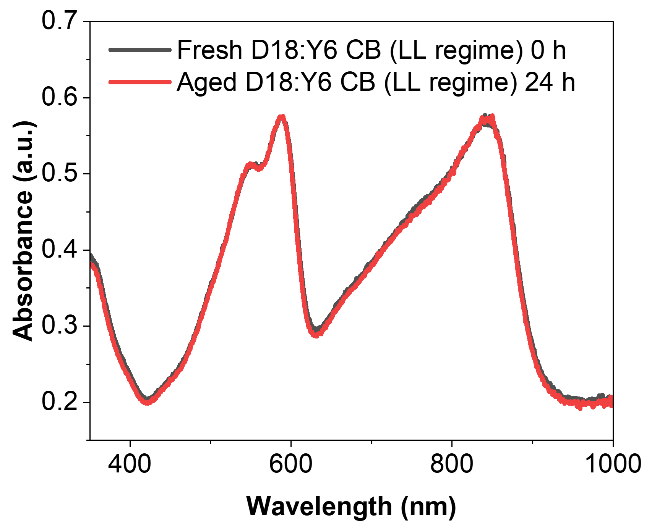


**Figure S16.** UV-Vis spectra of D18:Y6 CB blend films coated in the LL regime before and after light aging for 24 hours in the glovebox with N2 atmosphere.


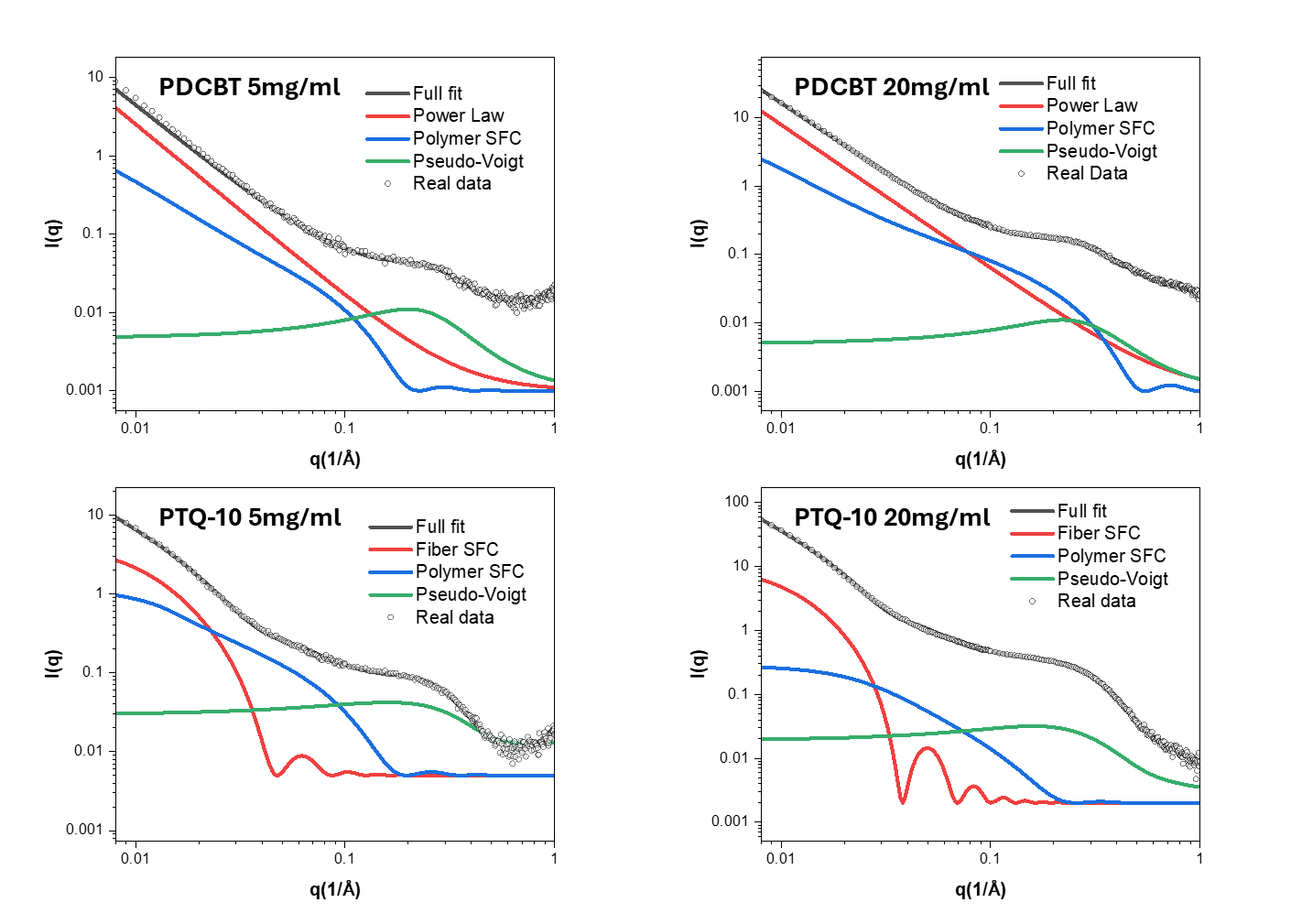


**Figure S17:** SAXS profiles and fitting deconvolution of PDCBT in 5 mg/mL and 20 mg/mL solutions. The Guinier region power law exponent of PDCBT and PTQ-10 was about -2 and -1.5, respectively.

**Table S3:** SAXS fitting parameters for PDCBT

| **Parameters** | **Values (PDCBT, 5mg/mL)** | **Values (PDCBT, 20mg/mL)** | **Values (PTQ-10, 5mg/mL)** | **Value (PTQ-10, 20mg/mL)** |
| --- | --- | --- | --- | --- |
| *L_1_* (nm) | 100* | 100* | 50* | 50* |
| *L_p1_* (nm) | 18* | 21.6 ± 6.0 | 24.2 ± 3.8 | 3.7 ± 0.38 |
| *R_1_* (Å) | 16.7 ± 1.9 | 7.3 ± 8.9 | 19.6 ± 0.5 | 15.3 ± 0.5 |
| *q_1_* (Å^-1^) | 0.2* | 0.22* | 0.16* | 0.16* |
| *FWHM_1_* | 0.258 ± 0.016 | 0.316 ± 0.008 | 0.290 ± 0.006 | 0.307 ± 0.007 |
| *w_1_* | 0 | 1 | 0 | 0.6 |
| *L_2_ (nm)* |  |  | 100* | 100* |
| *L_p2_ (nm)* |  |  | >>R_2_ | >>R_2_ |
| *R_2_ (nm*) |  |  | 8.12 ± 0.14 | 10.06 ± 0.14 |
| *Polydispersity* | 5.01 | 5.01 | 1.76 | 1.76 |

**Figure S18:** External Quantum Efficiency (EQE) spectrum of champion D18:Y6 cell. Maximum EQE > 70%, integrated Jsc ~ 21 mA/cm^2^.

**References**

[1] N. Schopp, S. Sabury, T. Chaney, J. Zhang, H. Wakidi, B. M. Kim, R. Sankar, H. M. Luong, P. Therdkatanyuphong, V. V. Brus, S. Marder, M. F. Toney, J. R. Reynolds, T.-Q. Nguyen, *ACS Energy Lett.* **2023**, *8*, 3307.

[2] L. Yang, J. Liu, S. Chodankar, S. Antonelli, J. DiFabio, *J. Synchrotron Radiat.* **2022**, *29*, 540.

[3] L. Yang, S. Antonelli, S. Chodankar, J. Byrnes, E. Lazo, K. Qian, *J. Synchrotron Radiat.* **2020**, *27*, 804.

[4] K. S. Park, Z. Xue, B. B. Patel, H. An, J. J. Kwok, P. Kafle, Q. Chen, D. Shukla, Y. Diao, *Nat. Commun.* **2022**, *13*, 2738.

[5] J. Ilavsky, *J. Appl. Crystallogr.* **2012**, *45*, 324.

[6] S. D. Oosterhout, V. Savikhin, J. Zhang, Y. Zhang, M. A. Burgers, S. R. Marder, G. C. Bazan, M. F. Toney, *Chem. Mater.* **2017**, *29*, 3062.
